# Translation, validity, and reliability of the European Portuguese version of the Touch Experiences and Attitudes Questionnaire

**DOI:** 10.7717/peerj.14960

**Published:** 2023-04-03

**Authors:** Ana Rita Pereira, Joana Antunes, Paula D. Trotter, Francis McGlone, Alberto J. González-Villar, Adriana Sampaio

**Affiliations:** 1 Psychological Neuroscience Laboratory (PNL), Research Center in Psychology (CIPsi), School of Psychology, Universidade do Minho, Braga, Portugal; 2 Research Centre for Brain and Behaviour, Liverpool John Moores University, Liverpool, United Kingdom

**Keywords:** TEAQ, Positive touch, Affective touch, Interpersonal touch, Social touch, Psychometric properties, Validation

## Abstract

**Background:**

Positive touch experiences have proved to be extremely important throughout our lifespan, with cascading effects on our social life. However, few questionnaires are available to measure attitudes and experiences of touch in the Portuguese population. This study aimed to translate and validate the European Portuguese version of the Touch Experiences and Attitudes Questionnaire (TEAQ), as a reliable and valid instrument to measure different aspects of affective touch experiences and attitudes.

**Methods:**

Therefore, an online sample of 384 (299 females and 85 males) participants, aged between 18 and 75 years (*M* = 24.59; *SD* = 9.56) was collected. Multidimensional Rasch model and confirmatory factor analysis were carried out, and also reliability and convergent and discriminant validity were determined. In addition, we examined sex differences in attitudes and experiences of touch.

**Results:**

Results showed good fit indexes for the 52-item six-factor model structure (friends and family touch, current intimate touch, childhood touch, attitudes to self-care, attitudes to intimate touch, and attitudes to unfamiliar touch). This instrument also showed good reliability and acceptable convergent and discriminant validity. Significant sex differences were found, with female participants reporting more positive touch experiences (including childhood touch, friends and family touch, and current intimate touch) and a more favourable attitude to self-care, with males showing a more positive attitude towards unfamiliar touch. Regarding attitudes towards the intimate touch, scores for both groups were comparable.

**Conclusion:**

Overall, the European Portuguese version of the TEAQ presented good psychometric properties and appears to be a reliable and valid self-report measure, being a useful and beneficial instrument in research and clinical settings.

## Introduction

Touch is an essential human need and plays a fundamental role in our social, emotional, and physical well-being throughout our entire life (Olausson et al., 2010). This sensory modality is fundamental from the early stages of life and plays an important role in the development of social relationships (Duhn, 2010; Gordon et al., 2010; Morrison, Löken & Olausson, 2010; Moszkowski & Stack, 2007). It is the first communication channel to develop at the ontological level, and probably the most relevant route for the formation of affective attachment (Cascio, Moore & McGlone, 2019; Miguel, Gonçalves & Sampaio, 2020). Additionally, touch plays a protective factor for children, mitigating stress (Feldman, Singer & Zagoory, 2010; Stack & Muir, 1990) and social isolation response (Perkeybile & Bales, 2015). Touch can also have a neurodevelopment effect in children, provoking stronger brain activation in premature infants who have been involved in supportive touch experiences, leading to improvements in cognitive, motor, and language competencies (Maitre et al., 2017). Further evidence demonstrates the importance of positive touch experiences throughout the lifespan, shaping social reward, attachment, communication, and emotional regulation (Cascio, Moore & McGlone, 2019).

Affective touch has been defined as tactile processing with a hedonic or emotional component (Morrison, 2016). This positive affective dimension of somatosensation is hypothesised to be primarily encoded by C-tactile afferents (CTs), a class of unmyelinated low-threshold mechanoreceptors that innervate the hairy skin of the body and have an optimised response to comforting and caress-like touch (Nordin, 1990; Olausson et al., 2010; Vallbo et al., 1993; Wessberg et al., 2003). Therefore, this system represents a specific neurobiological substrate for the rewarding properties of touch (McGlone, Wessberg & Olausson, 2014; Morrison, Löken & Olausson, 2010; Olausson et al., 2002). Information conveyed by these receptors is transmitted to second-order projection neurons of the dorsal horn of the spinal cord (Marshall, 2022), following pathways that ultimately target emotion-related areas like the posterior insular cortex (Olausson et al., 2002).

While evidence indicates that the bottom-up mechanisms of affective touch rely on the role of CTs, top-down factors can also affect the perception of touch experiences. The literature suggests that expectations and contextual cues relating to the hedonic value of touch can modify somatosensory processing (Ellingsen et al., 2016). In this line, the role of oxytocin stands out, since is related to decreased stress responses and can increase the prominence of socially relevant cues, or promote approach behaviour in social interactions, thus also being context-dependent (Cascio, Moore & McGlone, 2019; Kemp & Guastella, 2011; Shamay-Tsoory et al., 2009; Walker et al., 2017). The perception of touch can also be affected by sex, with females reportedly having more positive touch experiences throughout their lives (Benenson, Morash & Petrakos, 1998; Berghout Austin & Braeger, 1990; Upenieks & Schafer, 2022; Webb & Peck, 2015), and tend to perceive these experiences as more pleasant during their lifespan (Croy et al., 2019; Jönsson et al., 2017; Russo, Ottaviani & Spitoni, 2020). Comfort with social touch also depends on the kind of relationship we have with the other person (Suvilehto et al., 2015).

Abnormal processing or deprivation of social touch can be associated with a wide range of contextual factors. For example, childhood maltreatment is related to low ratings of touch valence and a preference for greater interpersonal distancing (Croy et al., 2016; Maier et al., 2020), while caregiver’s touch deprivation (or avoidance) is related to an increased risk for sensory processing problems (Lin et al., 2005; Wilbarger et al., 2010). Furthermore, there is also evidence that deprivation and unsatisfactory levels of social contact are associated with a greater occurrence of depression (Cochrane, 1990; Debrot et al., 2021). Altered touch sensitivity has also been observed in several neurodevelopmental conditions, with the case of Autism Spectrum Disorder (ASD) being of particular interest, since hypo- or hyper-sensitivity and avoidance of social contact are frequently reported symptoms (Cascio, Lorenzi & Baranek, 2016; Foss-Feig, Heacock & Cascio, 2012; Hyman et al., 2020; Kaiser et al., 2016). Finally, anhedonia towards affective touch has been observed in other disorders, such as anorexia nervosa or fibromyalgia (Boehme et al., 2020; Crucianelli et al., 2016), which emphasises the need for tools capable of reliably quantifying this sensory modality.

Currently, there are few tools that allow the evaluation of experiences or attitudes towards touch in the Portuguese population. One exception is the Social Touch Questionnaire (STQ) (Wilhelm et al., 2001), translated and validated for the Portuguese population by Vieira and colleagues (Vieira et al., 2016). The STQ measures perception, behaviours, and attitudes towards the social touch, with a focus on social interaction problems. Beyond revealing adequate reliability, the total scores of the STQ were positively correlated with the anxiety and avoidance subscales of the Social Interaction and Performance Anxiety and Avoidance Scale (SIPAAS) (Pinto-Gouveia et al., 2006). Despite its potential as a screening tool, there is a need for a test that assesses other relevant tactile dimensions, such as intimate touch and touch directed to oneself.

The Touch Experiences and Attitudes Questionnaire (TEAQ) (Trotter et al., 2018a) was developed to measure current touch experiences and previous experiences during childhood, as well as attitudes towards touch, thereby providing a holistic measure of touch experiences. The original version of TEAQ has an established and validated six-factor structure, covered by 57 items, namely Friends and Family Touch (FFT), Current Intimate Touch (CIT), Childhood Touch (ChT), Attitude to Self-Care (ASC), Attitude to Intimate Touch (AIT), and Attitude to Unfamiliar Touch (AUT). Its validation evidenced a good internal consistency (Cronbach’s *α* ranged from .81 to .93), construct validity in terms of discriminant validity, known-group validity, and convergent validity, and criterion validity in terms of predictive and concurrent validity. In addition to the original version, a short-form version of TEAQ was also validated for the Russian population (Trotter et al., 2018b). Composed of 37 items, the short-form version revealed a reliable and validated model structure of five-factors, covering the dimensions of Attitude to Friendly Touch (AFT), ChT, ASC, CIT, and AIT (Cronbach’s *α* ranged from .83 to .88), not reproducing the original dimension of AUT. It was also proposed the possibility to calculate a total score for this short-form version, through the sum of the subscale scores, which also revealed high reliability (Cronbach’s *α* = .92). Recently, a 55 items Mongolian version was also validated (Tumurbaatar et al., 2022), which revealed a reliable and validated model structure of six-factors as the original version. Internal consistency of this version was well supported, with Cronbach’s *α* ranging from .68 to .90.

To overcome the absence of a self-report measure that assesses current and previous experiences and attitudes towards touch over a diverse number of domains, the present study aimed to translate and validate the original English version of the TEAQ (Trotter et al., 2018a) for the European Portuguese population. For this purpose, we will test the six-factor model structure by performing a multidimensional Rasch model and a confirmatory factor analysis and by examining the reliability of the questionnaire, expecting a robust factor structure. Furthermore, we will assess convergent validity through the STQ (Vieira et al., 2016), as used in the original version, and we will assess discriminant validity through the NEO Five-Factor Inventory (NEO-FFI) (Magalhães et al., 2014), as in the Russian validation. We will also evaluate sex differences, expecting that female participants will have better attitudes towards touch, especially in dimensions such as ASC and FFT, as described in previous works (Trotter et al., 2018a; Trotter et al., 2018b; Tumurbaatar et al., 2022). The validation of the European Portuguese version of TEAQ will provide a new tool with potential use in the clinical and research contexts.

## Materials & Methods

### Participants

A total of 384 participants completed the study, 299 were female (77.9%) and 85 (22.1%) were male. Age ranged from 18 to 75 years (*M* = 24.59; *SD* = 9.56). Demographic variables are described in Table 1. Inclusion criteria included being 18 years of age or older, being native European Portuguese speakers, and completing all questionnaires (TEAQ, STQ, and NEO-FFI). From an initial sample of 475, 73 participants were excluded for not completing all questionnaires, and 18 for not being native European Portuguese speakers. All the participants voluntarily agreed to answer the questionnaire and provided written informed consent. None of the participants received any monetary compensation, and 67 university students received credits for their participation. The study protocol obtained approval from the ethics committee for Research in Human and Social Sciences of the University of Minho (CEICSH 023/2020) and was in accordance with the Declaration of Helsinki.

**Table 1:** Sociodemographic characteristics of the sample (N = 384).

	**Total Sample (N = 384)**	**Men (*n* = 85)**	**Women (*n* = 299)**
	*M* **(SD)**	*M* **(SD)**	*M* **(SD)**
**Age**	24.59 (9.56)	27.82 (12.57)	23.67 (8.31)
	**n (%)**	**n (%)**	**n (%)**
**Education level (in years)**			
≤ 4	1 (0.3)	1 (1.2)	–
5–11	211 (54.9)	40 (47.1)	171 (57.2)
≥ 12	172 (44.8)	44 (51.8)	128 (42.8)
**Civil status**			
Single	268 (69.8)	54 (63.5)	214 (71.6)
In a relationship	86 (22.4)	20 (23.5)	66 (22.1)
Married	25 (6.5)	10 (11.8)	15 (5)
Divorced/Widow	5 (1.3)	1 (1.2)	4 (1.3)
**Professional situation**			
Student	275 (71.6)	48 (56.5)	227 (75.9)
Full time employee	61 (15.9)	21 (24.7)	40 (13.4)
Part-time employee	7 (1.8)	2 (2.4)	5 (1.7)
Unemployed/Retired/Other	41 (10.7)	14 (16.5)	27 (9)
**Household**			
1	19 (4.9)	9 (10.6)	10 (3.3)
2–4	338 (88)	69 (81.2)	269 (90)
>4	27 (7)	7 (8.3)	20 (6.7)
**Children**			
0	352 (91.7)	75 (88.2)	277 (92.6)
1	12 (3.1)	3 (3.5)	9 (3)
≥ 2	20 (5.2)	7 (8.2)	13 (4.3)


### Measures

### Sociodemographic information

Participants completed a sociodemographic questionnaire assessing personal information, namely age, biological sex, nationality, educational level, civil status, number of people they live with, number of children, and professional situation.

### Touch Experiences and Attitudes Questionnaire (TEAQ)

The TEAQ (Trotter et al., 2018a) is a 57-items self-report questionnaire that reflects touch experiences at present and during childhood, as well as attitudes towards touch. The original version (UK validated) includes six subscales: Friends and Family Touch (FFT; with 11 items), relating to interpersonal touch experiences with friends and family; Current Intimate Touch (CIT; with 14 items), determines current levels of intimate touch experiences between people who are emotionally close or in a romantic relationship; Childhood Touch (ChT; with nine items), provides a measure of positive childhood touch experiences; Attitude to Self-Care (ASC; with five items), related to skin care and grooming behaviours associated with positive self-care; Attitude to Intimate Touch (AIT; with 13 items), referring to attitudes towards touch experiences between people who are emotionally close or in a romantic relationship; and Attitude to Unfamiliar Touch (AUT; with five items), that relates to attitudes to touch experiences with someone that the individual is less close to, or even a stranger. Items are answered using a 5-point Likert scale ranging from 1—‘Disagree strongly’, 2—‘Disagree a little’, 3—‘Neither agree nor disagree’, 4—‘Agree a little’ to 5—‘Agree strongly’, with eight reverse-scored items. Therefore, higher scores reflect a more positive attitude toward touch or more frequent experiences. The total score for each subscale is obtained by calculating the mean score of the items for each subscale.

### Social Touch Questionnaire (STQ)

The STQ (Wilhelm et al., 2001; Portuguese version, Vieira et al., 2016) is a 20-item self-report questionnaire that measures individual perceptions, behaviours, and attitudes toward the social touch. The items represent a vast variety of social interactions, such as touching *versus* being touched, touching someone you meet *versus* touching a stranger, touching someone in a public place *versus* in a private place, and touching without a sexual connotation *versus* touching with a sexual connotation. Each item is rated on a 5-point Likert scale to state how far the statements are true, ranging from 0 (‘Not at all’) to 4 (‘Extremely’). The score ranges from 0 to 80 and results from the sum of the items, in which a low score represents the lowest avoidance of social touch, and a high score represents the most avoidance of social touch. The Portuguese version demonstrated good internal consistency (Cronbach’s *α* = .73). In the present study, this questionnaire had Cronbach’s *α* = .82.

### NEO Five-Factor Inventory (NEO-FFI)

The NEO-FFI (McCrae & Costa, 1987; Portuguese version, Magalhães et al., 2014) is a 60-item self-report inventory that assesses individual personality traits. The NEO-FFI is a short version of NEO PI-R and provides a concise measure of the five basic personality factors (Neuroticism, Extraversion, Openness to Experience, Agreeableness, and Conscientiousness) with 12 items for each factor. Each item is measured on a 5-point Likert scale of agreement (0—‘Strongly disagree’, 1—‘Disagree’, 2—‘Neither agree nor disagree’, 3—‘Agree’, 4—‘Strongly agree’). The score for each personality factor results from the sum of the respective 12 items. The Portuguese version demonstrated good internal consistency (with Cronbach’s alpha values ranging from .71 to .81). In our study it had Cronbach’s alpha values between .66 and .85.

### Procedure

**Figure 1:**
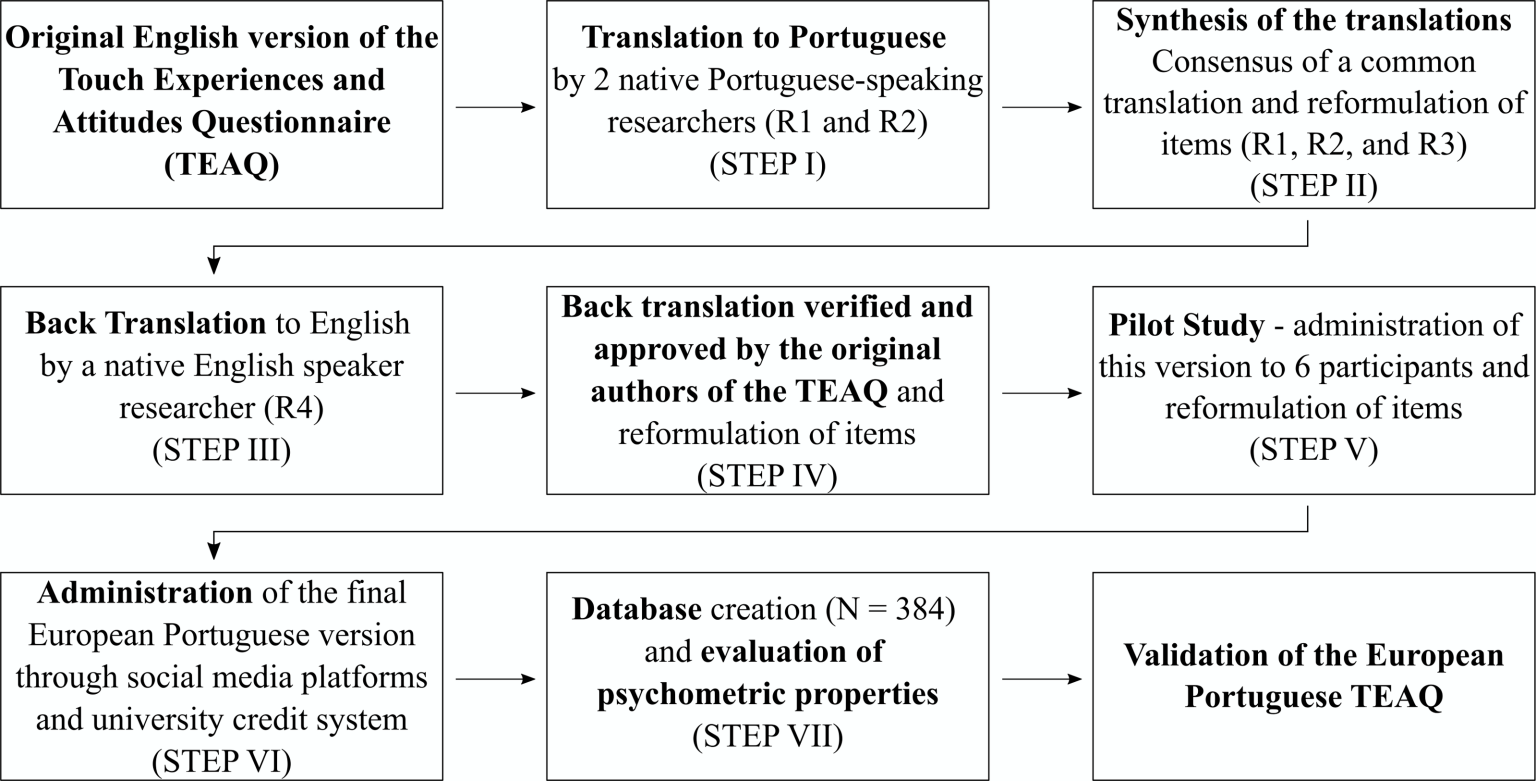
Flowchart of the European Portuguese adaptation of the TEAQ.

The translation of the original English version of the TEAQ to European Portuguese was performed following previous recommendations (see Fig. 1) (Hilton & Skrutkowski, 2002). First, the original version of the TEAQ was translated to European Portuguese independently by two translators (psychology researchers), whose native language is European Portuguese. After that, a reconciled version was made by three researchers that compared the two versions of the translation and the original one and built a unique version. This version was back-translated from European Portuguese into English by a bilingual translator (psychology researcher), whose native language is English, and without any knowledge of the original items of the TEAQ. To analyse the equivalence between the original version and the retroversion, we also consulted the original authors. A preliminary version of the European Portuguese TEAQ was developed, and a pilot test was conducted with six individuals, three male and three female, aged between 22 and 25 ( *M* = 23.83; *SD* = 1.07), to verify any difficulty or doubt when completing the items, and based on their feedback, a final version was developed and released (no changes were made). Participants were recruited *via* social media platforms and university credit system, through which they were asked to complete an online questionnaire conducted *via* Qualtrics software (Qualtrics, Provo, UT). All data were collected between April 2020 and January 2021. The final European Portuguese version of the TEAQ is included in Appendix A. The authors have permission to use this instrument from the copyright holders (Trotter et al., 2018a).

### Statistical analysis

The descriptive and inferential statistical analyses were conducted using IBM SPSS 28. The multidimensional Rasch model and the differential item functioning (DIF) were estimated using the ACER Conquest 5.19.5 software. Confirmatory factor analysis was carried out using IBM SPSS Amos™ 28.

To assess normality, we analysed the asymmetry values, skewness and kurtosis, revealing no significant biases in relation to the averages, with values lower than —3— and —10— for skewness and kurtosis, respectively (Kline, 2016). Also, to examine the suitability for conducting a confirmatory factor analysis (CFA), the factorability of the correlation matrix was explored. Bartlett’s sphericity test proved to be statistically significant (*p* < .001) and the Kaiser-Meyer-Olkin (KMO) value was .90, above the recommended criterion of .60 (Pallant, 2011). An analysis of the correlation matrix also revealed several correlation coefficients greater than .30 (Tabachnick & Fidell, 2013).

The parameters of the multidimensional Rasch model were estimated with a marginal maximum likelihood estimation method for item parameters and a Monte Carlo-based approach with 2000 nodes for person parameters. Through this model, we obtained information about item fit, with the scores of the infit and outfit mean square (MNSQ) between .60 and 1.4 indicating that the item was fit for the model with Likert scales (Wright & Linacre, 1994). Items outside this range were removed from the questionnaire. Additionally, the performance of the 5-point Likert scale was examined using an outfit MNSQ <2.0, demonstrating that the response category was functioning well (Linacre, 1999). Furthermore, we examined the item separation reliability, in which values above .67 were considered acceptable (Utari, Liliawati & Utama, 2021). Finally, we obtained information about person separation reliability for each TEAQ subscale in the form of plausible values (PV) reliability indices, in which values above .70 were considered acceptable (Fauth et al., 2019).

The original six-factor model and the parcelled six-factor model of the TEAQ, as originally proposed by Trotter and colleagues (Trotter et al., 2018a), were tested through a CFA, according to the maximum likelihood method. The parcelled six-factor model was used since the reliability of structural equation modelling is reduced when there are a large number of variables. Using the item parcelling method (Nasser & Wisenbaker, 2003) to produce three measures for each factor, it was determined whether the model fit indexes would be affected, as suggested by Yang, Nay & Hoyle (2010). In this procedure, the same item parcelling suggested by Trotter et al. (2018a) in the original version of this questionnaire was used, excluding only the items removed in the multidimensional Rasch model analysis. Thus, we explored if the parcelling method improved the criteria for a good model. The following indexes were considered to determine the goodness of model fit: Relative Chi-Square (x2/df), Normed Fit Index (NFI), Comparative Fit Index (CFI), Goodness-of-Fit Index (GFI), and Root Mean Square Error of Approximation (RMSEA). We considered values of Relative Chi-Square ≤ 5 acceptable fit and ≤ 2 perfect fit; NFI values ≥ .90 acceptable fit and ≥ .95 good fit; CFI values ≥ .90 acceptable fit, ≥ .95 good fit, and ≥ .97 perfect fit; GFI values between .85 and .89 acceptable fit, ≥ .90 good fit, and ≥ .95 perfect fit; and RMSEA values between .10 and .08 weak fit, ≤ .08 good fit, and ≤ .06 perfect fit. The Akaike Information Criterion (AIC) was also examined to compare models (Arbuckle, 2013; Hooper, Coughlan & Mullen, 2008).

Additionally, internal consistency was assessed through Cronbach’s alpha values, with values equal to or greater than .70 considered satisfactory (Bland & Altman, 1997; Streiner, 2003). We also computed Pearson’s correlations between TEAQ subscales, and between TEAQ and the other questionnaires, namely the STQ (for convergent validity), and the NEO-FFI (for discriminant validity) (*r* ≤ —.12——very weak correlation; —.12—≥ *r* ≥—.24——weak correlation; —.24—≥*r* ≥—.41——moderate correlation; —.41—≥ *r* ≥ —1——high correlation) (Lovakov & Agadullina, 2021). DIF was explored using the Rasch model method to compare differences in the functioning of individual items across the two groups (female and male participants), with the following criteria: DIF contrast <.50 logits—negligible DIF; DIF contrast between .50 and 1 logits—moderate DIF; and DIF contrast >1 logits—substantial; if the DIF was substantial and significant it might mean that each group interprets the scale differently and that confounding factors are influencing that interpretation (Bond & Fox, 2015; Cameron et al., 2014). To examine sex differences in the TEAQ subscales, independent samples *t*-tests were performed, and effect-sizes were measured through Cohen’s *d* calculation (Cohen’s *d*: ≥ .20—small; ≥ .50—medium; ≥ .80—large) (Cohen, 1992).

## Results

### Multidimensional Rasch model analysis

**Figure 2:**
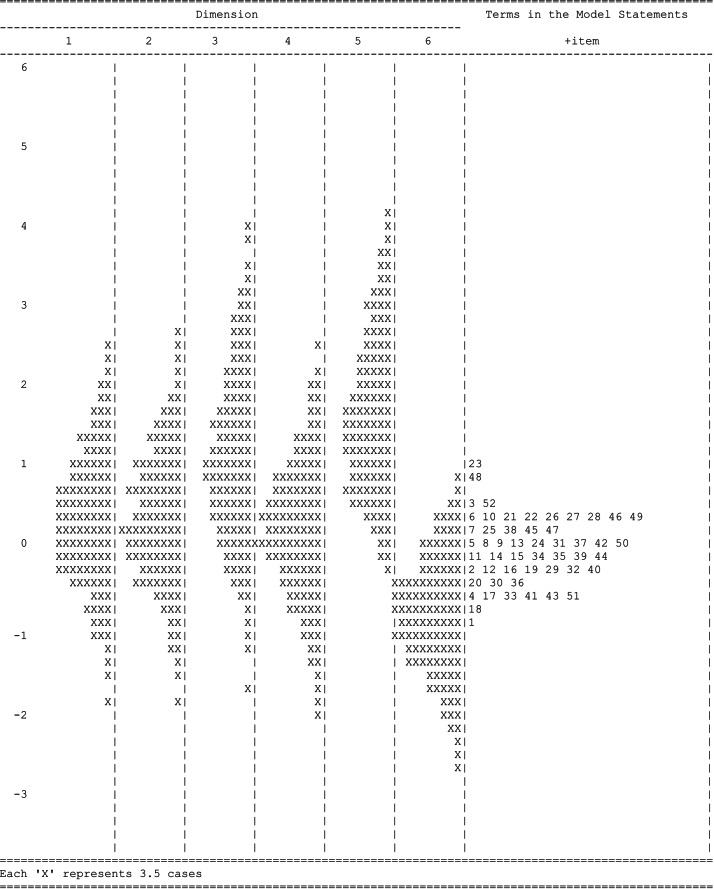
Wright map of TEAQ (52-items). Dimension 1 = FFT; Dimension 2 = CIT; Dimension 3 = ChT; Dimension 4 = ASC; Dimension 5 = AIT; Dimension 6 = AUT.

Table 2 shows the statistics for the TEAQ items, including item difficulty location (endorsability) and item fit statistics. All items fit the multidimensional Rasch rating scale model, with the values of infit and outfit MNSQ within the acceptable range (.60 –1.4). The location of items is in the range of −0.842 (Q1) to 1.020 (Q23) logits with an item separation reliability of .99. Item 1 (“Eu não gosto quando as pessoas são muito afetuosas fisicamente em relação a mim”. [I dislike people being very physically affectionate towards me]) was the easiest to endorse (*i.e.,* the easiest item for obtaining a score of 5) with its location at −0.842 logits, and item 23 (“Eu costumo tomar um duche ou um banho com alguém” [I often take a shower or bath with someone]) was the most difficult to endorse with its location at 1.020 logits. Five items (three items from the CIT subscale - items 23, 53, and 54 from the original version; one item from the ChT subscale - item 42 from the original version; and one item from the AIT subscale - item 20 from the original version) were excluded since the infit and outfit MNSQ values were not within the acceptable range. The item’s threshold (*τ*) appeared in the analysis from the lowest to the highest (*τ*1 = −0.921; *τ*2 = −0.177; *τ*3 = −0.064; *τ*4 = 1.161). Additionally, person separation reliability in the form of PV reliability for each subscale were .97 for FFT, .93 for CIT, .93 for ChT, .82 for ASC, .87 for AIT, and .82 for AUT, all with acceptable criteria. The distribution of persons and the 52 items’ threshold estimates on the different TEAQ subscales can be consulted in the wright map (see Fig. 2). Therefore, after the multidimensional Rasch model analysis, the Portuguese TEAQ was left with 52 items: FFT (11 items)—4, 13, 14, 16, 20, 28, 36, 45, 48, 51, and 52; CIT (11 items)—11, 17, 18, 23, 25, 27, 34, 39, 42, 43, and 46; ChT (8 items)—5, 6, 9, 15, 21, 30, 31, and 33; ASC (5 items)—2, 7, 40, 49, and 50; AIT (12 items)—8, 10, 12, 19, 22, 24, 29, 32, 38, 41, 44, and 47; AUT (5 items)—1, 3, 26, 35, and 37; in which 1, 3, 9, 26, 35, and 37 are reverse-scored items.

**Table 2:** Item measures and fit statistics for the TEAQ (52-items).

**Item**	**Estimate**	**SE**	**Outfit MNSQ**	**Infit MNSQ**
**Factor 1: FFT**				
Q4	−0.664	0.039	1.39	1.31
Q12	−0.242	0.049	0.78	0.86
Q13	0.144	0.037	0.99	1.00
Q15	−0.156	0.047	0.93	0.84
Q19	−0.205	0.049	1.26	1.10
Q27	0.381	0.037	1.34	1.23
Q43	−0.599	0.039	0.82	0.83
Q46	0.380	0.119	1.35	1.26
Q49	0.332	0.037	1.28	1.23
Q50	0.003	0.074	1.13	1.19
**Factor 2: CIT**				
Q10	0.492	0.044	1.17	1.09
Q16	−0.170	0.037	0.94	0.89
Q17	−0.511	0.038	1.08	1.11
Q22	0.453	0.045	0.91	0.75
Q24	0.016	0.047	1.16	1.11
Q26	0.476	0.040	0.91	0.96
Q33	−0.596	0.121	0.76	0.83
Q37	0.001	0.077	0.73	0.73
Q40	−0.272	0.037	0.93	0.92
Q41	−0.610	0.052	0.60	0.72
Q44	−0.038	0.048	0.75	0.78
**Factor 3: ChT**				
Q5	0.152	0.045	0.72	0.66
Q6	0.365	0.044	0.90	0.89
Q14	−0.141	0.037	0.96	0.97
Q20	−0.397	0.038	0.74	0.80
Q29	−0.302	0.049	0.79	0.84
Q30	−0.382	0.048	1.31	1.31
Q32	−0.228	0.049	1.00	0.88
Q38	0.288	0.046	0.95	0.86
**Factor 4: ASC**				
Q2	−0.292	0.037	0.89	0.90
Q7	0.230	0.037	0.83	0.83
Q39	−0.063	0.037	0.92	1.00
Q47	0.237	0.158	0.74	0.71
Q48	0.928	0.037	1.18	1.15
**Factor 5: AIT**				
Q8	0.138	0.046	1.20	1.11
Q9	0.152	0.045	1.00	0.97
Q11	−0.131	0.037	1.12	1.14
Q18	−0.780	0.039	1.15	1.14
Q21	0.420	0.044	1.20	1.19
Q23	1.020	0.037	1.39	1.37
Q28	0.374	0.037	0.78	0.80
Q31	0.045	0.046	0.65	0.66
Q36	−0.390	0.038	0.65	0.62
Q42	0.153	0.037	0.71	0.71
Q45	0.273	0.037	1.08	0.99
**Factor 6: AUT**				
Q1	−0.842	0.037	1.05	1.02
Q3	0.513	0.040	1.08	1.07
Q25	0.314	0.037	1.18	1.16
Q34	−0.163	0.037	1.25	1.24
Q35	−0.148	0.037	0.74	0.75

**Notes.**

SE standard error MNSQ mean square

### Confirmatory factor analysis and internal consistency

**Figure 3:**
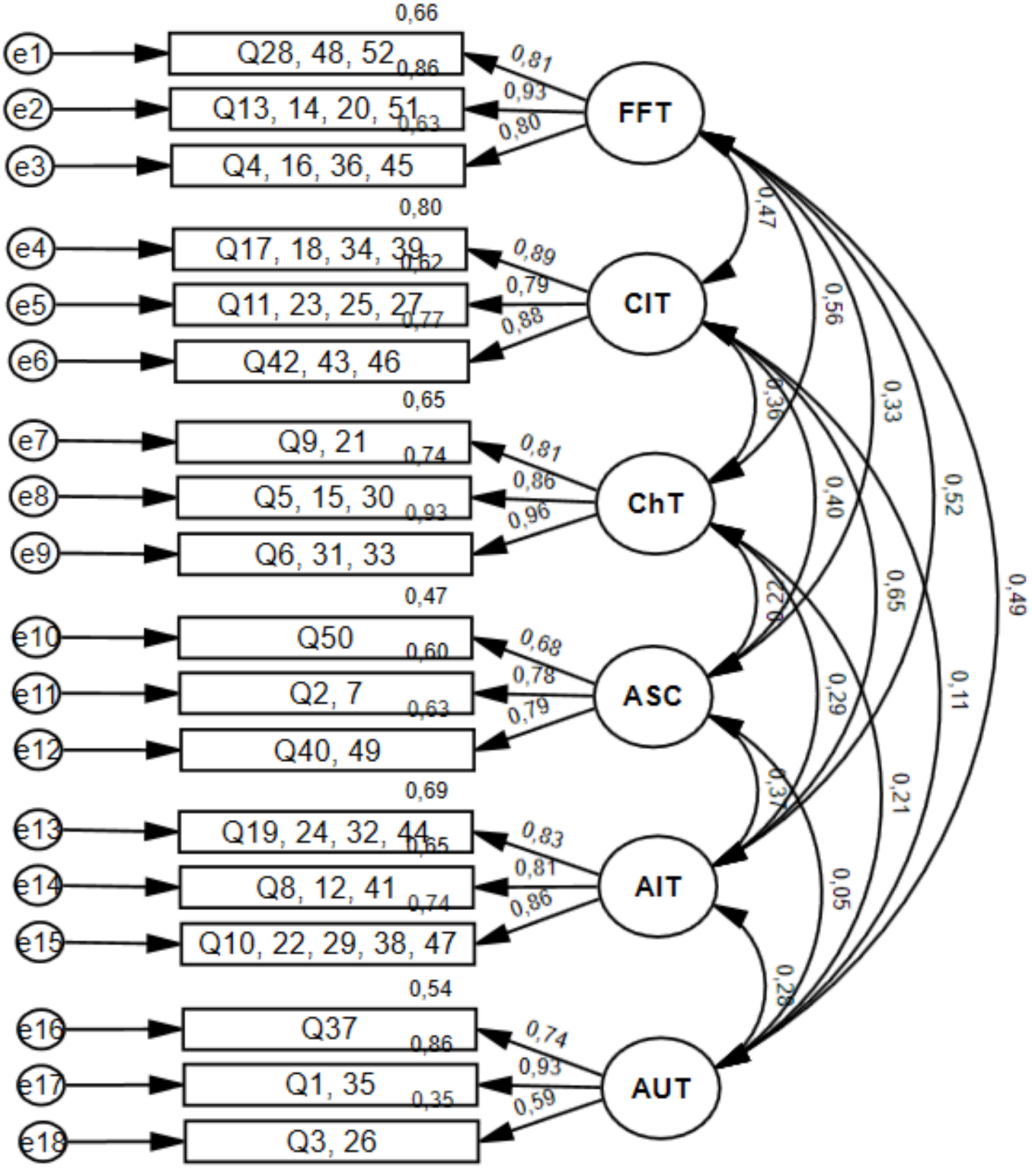
Confirmatory factor analysis for the parcelled six-factor model of TEAQ (52-items).

Results of the 52-items confirmatory factor analyses for the original and the parcelled six-factor model are reported in Table 3. Examination of the goodness of fit indexes of competing models revealed that the parcelled six-factor model had a good fit to data (see Table 3 and Fig. 3), as observed by the authors of the original version of the TEAQ (Trotter et al., 2018a). The six factors of the TEAQ revealed good to excellent values of internal consistency, with the exception of AUT subscale, which revealed acceptable internal consistency (FFT, *α* = .90; CIT, *α* = .88; ChT, *α* = .90; ASC, *α* = .81; AIT, *α* = .89; AUT, *α* = .78).

### Correlations between subscales

All TEAQ subscale scores correlated significantly with each other, except the AUT with the CIT and ASC subscales (see Table 4). Significant high correlations were found between FFT and CIT (*r* = .42; *p* < .001), FFT and ChT (*r* = .50; *p* < .001), FFT and AIT (*r* = .46; *p* < .001), and CIT and AIT (*r* = .58; *p* < .001) subscales. Significant moderate correlations were found between FFT and ASC (*r* = .30; *p* < .001), FFT and AUT (*r* = .40; *p* < .001), CIT and ChT (*r* = .32; *p* < .001), CIT and ASC (*r* = .34; *p* < .001), ChT and AIT (*r* = .26; *p* < .001), and ASC and AIT (*r* = .31; *p* < .001) subscales. Significant weak correlations were found between ChT and ASC (*r* = .21; *p* < .001), ChT and AUT (*r* = .15; *p* < .01), and AIT and AUT (*r* = .19; *p* < .001) subscales. The strongest correlation was observed between CIT and AIT (*r* = .58), and the weakest significant correlation between ChT and AUT (*r* = .15).

### Correlations with other scales: convergent and discriminant validity

Regarding convergent validity, the STQ and TEAQ subscales correlations were significant (*p* < .001). These results revealed high correlations with FFT (*r* =  − .59), AIT (*r* =  − .43), and AUT (*r* =  − .70) subscales, moderate correlations with CIT (*r* =  − .25), and ChT (*r* =  − .31) subscales, and a weak correlation with ASC (*r* =  − .19) subscale (see Table 5). Concerning discriminant validity, we made correlations between the five personality factors of NEO-FFI and the TEAQ subscales (see Table 5). For the Neuroticism (N) factor, a moderate correlation was found with the AUT (*r* =  − .29; *p* < .001) subscale, and weak correlations with the FFT (*r* =  − .16; *p* < .01), CIT (*r* =  − .12; *p* < .05), and ChT (*r* =  − .15; *p* < .01) subscales, but no significant correlations with ASC and AIT. For the Extraversion (E) factor, a high correlation emerged with the FFT (*r* = .45; *p* < .001) subscale, moderate correlations with the CIT (*r* = .35; *p* < .001), ChT (*r* = .30; *p* < .001), AIT (*r* = .35; *p* < .001), and AUT (*r* = .34; *p* < .001) subscales, and a weak correlation with the ASC (*r* = .15; *p* < .01) subscale. For the Openness to Experience (O) factor, we observed a moderate correlation with the AIT (*r* = .25; *p* < .001) subscale, and a very weak correlation with the FFT (*r* = .11; *p* < .05) subscale, but no significant correlations with the CIT, ChT, ASC, and AUT. For the Agreeableness (A) factor, moderate correlations were observed with FFT (*r* = .34; *p* < .001), ChT (*r* = .27; *p* < .001), and AIT (*r* = .32; *p* < .001) subscales, and weak correlations with the CIT (*r* = .21; *p* < .001), ASC (*r* = .14; *p* < .01), and AUT (*r* = .17; *p* < .001) subscales. Lastly, for the Conscientiousness (C) factor, a moderate correlation was found with the CIT (*r* = .25; *p* < .001) subscale, and weak correlations with the FFT (*r* = .19; *p* < .001), ChT (*r* = .16; *p* < .01), ASC (*r* = .19; *p* < .001), and AIT (*r* = .23; *p* < .001) subscales, but no correlation with AUT.

**Table 3:** Model fit indexes.

**Model**	*χ* ^**2**^	**df**	*χ* ^**2**^ **/df**	**NFI**	**CFI**	**GFI**	**RMSEA**	**AIC**
**Original**	3507,44	1261	2,78	.70	.78	.71	.07	3743,435
**Parcelled**	342,67	120	2,86	.92	.95	.91	.07	444,667

**Notes.**

*χ*^2^ Chi-Square df degrees of freedom NFI Normed Fit Index CFI Comparative Fit Index GFI Goodness-of-Fit Index RMSEA Root Mean Square Error of Approximation AIC Akaike Information Criterion

**Table 4:** Pearson’s *r* correlation coeficient between each TEAQ subscales.

	**FFT**	**CIT**	**ChT**	**ASC**	**AIT**	**AUT**
**Friends and family touch (FFT)**	–	.42^***^	.50^***^	.30^***^	.46^***^	.40^***^
**Current intimate touch (CIT)**	–	–	.32^***^	.34^***^	.58^***^	.09
**Childhood touch (ChT)**	–	–	–	.21^***^	.26^***^	.15^**^
**Attitude to self-care (ASC)**	–	–	–	–	.31^***^	.01
**Attitude to intimate touch (AIT)**	–	–	–	.-	–	.19^***^
**Attitude to unfamiliar touch (AUT)**	–	–	–	–	–	–

**Notes.**

*** *p* < .001

** *p* < .01

**Table 5:** TEAQ subscales correlations with STQ and NEO-FFI.

	**FFT**	**CIT**	**ChT**	**ASC**	**AIT**	**AUT**
**STQ**	−.59^***^	−.25^***^	-.31^***^	−.19^***^	−.43^***^	−.70^***^
**N (NEO-FFI)**	−.16^**^	−.12^*^	−.15^**^	.06	.00	−.29^***^
**E (NEO-FFI)**	.45^***^	.35^***^	.30^***^	.15^**^	.35^***^	.34^***^
**O (NEO-FFI)**	.11^*^	.03	.06	.09	.25^***^	.03
**A (NEO-FFI)**	.34^***^	.21^***^	.27^***^	.14^**^	.32^***^	.17^***^
**C (NEO-FFI)**	.19^***^	.25^***^	.16^**^	.19^***^	.23^***^	−.01

**Notes.**

STQ Social Touch Questionnaire N Neuroticism E Extraversion O Openness to Experience A Agreeableness C Conscientiousness FFT Friends and Family Touch CIT Current Intimate Touch ChT Childhood Touch ASC Attitude to Self-Care AIT Attitude to Intimate Touch AUT Attitude to Unfamiliar Touch

* *p* < .05

** *p* < .01

*** *p* < .001

### Sex differences on TEAQ

No substantial DIF was found in the TEAQ items across female and male participants. Moderate DIF was found for item 3—“Eu tenho de conhecer alguém relativamente bem para desfrutar do seu abraço”. [I have to know someone quite well to enjoy a hug from them] (*i.e.,* relatively easier for males than females [0.608 logits]), item 24—“Eu gosto de fazer sexo”. [I enjoy having sex] (*i.e.,* relatively easier for males than females [0.636 logits]), item 26—“Eu não gosto de ter proximidade física com pessoas que não conheço bem”. [I am put off by physical familiarity] (*i.e.,* relatively easier for males than females [0.622 logits]), item 37—“Faz-me sentir desconfortável se alguém que eu não conheço muito bem me toca de uma maneira amigável”. [It makes me feel uncomfortable if someone I don’t know very well touches me in a friendly manner] (*i.e.,* relatively easier for males than females [0.528 logits]), item 40—“Eu gosto de exfoliar a minha pele”. [I like exfoliating my skin] (*i.e.,* relatively easier for females than males [0.532 logits]), and item 50—“Eu gosto de usar máscaras faciais na minha pele”. [I like to use face masks on my skin] (*i.e.,* relatively easier for females than males [0.954 logits]). All other items have DIF <.50, and item 19—“Beijar é uma boa forma de expressar atração física”. [Kissing is a great way of expressing physical attraction] revealed the same level of difficulty for both groups. These findings show that, regardless of other variables, the items performed similarly for people with the same level of ability.

Table 6 shows the sex differences on TEAQ subscales. Females showed higher scores on FFT, CIT, ChT, and ASC subscales than males, with a large effect size on all the subscales. AIT subscale scores for female and male participants were comparable. AUT subscale scores were higher for males than females, with a large effect size.

**Table 6:** T-test results for sex diferences across TEAQ subscales.

	**Male**	**Female**	***t*** **(df)**	***p*** **-value**	**Cohen’s** ***d***	**95% CI**
	**(*n* = 85)**	**(*n* = 299)**				
	***M*** **(SD)**	***M*** **(SD)**				
**FFT**	3.15 (.89)	3.42 (.87)	−2.50 (382)	.01^*^	.88	−.549; -.065
**CIT**	3.13 (.86)	3.49 (.86)	-3,45 (382)	<.001^***^	.86	−.667; −.181
**ChT**	3.73 (.88)	3.96 (.89)	−2,06 (382)	.04^*^	.89	−.494; −.011
**ASC**	2.36 (.83)	3.52 (.89)	−10,79 (382)	<.001^***^	.87	−1.584; −1.067
**AIT**	4.20 (.63)	4.33 (.59)	−1,71 (382)	.09	.60	−.452; .031
**AUT**	2.62 (.87)	2.18 (.78)	4.48 (382)	<.001^***^	.80	.306; .794

**Notes.**

FFT Friends and Family Touch CIT Current Intimate Touch ChT Childhood Touch ASC Attitude to Self-Care AIT Attitude to Intimate Touch AUT Attitude to Unfamiliar Touch M mean SD standard deviation df degrees of freedom CI Confidence Interval

* *p* < .05

*** *p* < .001

## Discussion

The present study explored the psychometric properties of the 52-items European Portuguese version of the TEAQ. The original version proposes a six-factor structure (Trotter et al., 2018a), which also proved to be appropriate for the Portuguese version. A multidimensional Rasch model and a confirmatory factor analysis showed good fit index values for the 52-items six-factor model, demonstrating that all dimensions mentioned above can be assessed through this version of the questionnaire. Regarding instrument reliability, the Portuguese version of the TEAQ revealed high Cronbach’s *α* values, and overall, demonstrated good to excellent levels of internal consistency for the six factors individually.

All TEAQ subscales positively correlated with each other, except for the attitude to unfamiliar touch with current intimate touch and attitude to self-care, in which no significant correlation emerged. The weakest significant correlation observed was between childhood touch experiences and attitude to unfamiliar touch, and the strongest significant correlation was between current intimate touch and attitude to intimate touch. Despite cultural differences, these results are in line with the results found in the other available versions of the questionnaire (Trotter et al., 2018a; Trotter et al., 2018b). In similarity with our results, one of the weakest correlations found in the original version (Trotter et al., 2018a) was between attitude to unfamiliar touch and childhood touch experiences. The same applies to the strongest correlation between current intimate touch and attitude to intimate touch, being the strongest correlation in the UK version (Trotter et al., 2018a), and also one of the strongest correlations in the Russian version (Trotter et al., 2018b). Moreover, the factor of unfamiliar touch attitudes was the only factor that was not reproduced in the Russian short-form version (Trotter et al., 2018b), and is also globally our factor with the lowest values.

Concerning the validity of the questionnaire, acceptable convergent validity has been demonstrated with the STQ (Vieira et al., 2016) and all TEAQ subscales, in which the correlations were negative, being the attitude to unfamiliar touch factor the strongest correlation. This finding was expected since the STQ was developed to assess anxiety with social touch situations, including situations involving contact with strangers. Furthermore, the lowest correlations were with the CIT and ASC subscales, since they are dimensions not covered in the STQ. This is in accordance with the original version, in which the AUT subscale revealed the strongest correlation, and the ASC subscale revealed the lowest correlation. Moreover, acceptable discriminant validity was demonstrated with NEO-FFI (Magalhães et al., 2014), with most TEAQ subscales showing very weak, weak, or no significant correlation (19 out of 30; 63.33%) with the NEO-FFI factors, although there are some moderate correlations (10 out of 30; 33.33%) and a high correlation (3.33%). The strongest correlation was between the friends and family touch subscale and the extraversion factor, in accordance with the Russian version (Trotter et al., 2018b).

Regarding the effects of sex on experiences and attitudes towards touch, we found that females scored higher than males on the subscales about touch experiences at present and during childhood (FFT, CIT, and ChT subscales), suggesting that females experience more positive physical touch than males during their lifetime. These differences go in the same direction as those found in previous versions in the TEAQ (Trotter et al., 2018a; Trotter et al., 2018b). Moreover, there results are in line with previous research reporting that mothers tend to have a closer physical relationship with their daughters than with their sons (Benenson, Morash & Petrakos, 1998), and females have more positive touch experiences than males during adulthood (Webb & Peck, 2015), and in later life (Upenieks & Schafer, 2022). This greater involvement of females in touch experiences can be explained by their higher involvement in social activities (Upenieks & Schafer, 2022) and by their tendency to perceive these positive touch experiences as more pleasant compared to males during their lifespan (Croy et al., 2019; Jönsson et al., 2017; Russo, Ottaviani & Spitoni, 2020). Concerning attitude to self-care (ASC subscale), female participants also scored significantly higher than males, suggesting that females have a more positive attitude towards self-care, also in agreement with what was found in previous TEAQ versions (Trotter et al., 2018a; Trotter et al., 2018b; Tumurbaatar et al., 2022). Research has shown that females report their appearance to be more important than males (Quittkat et al., 2019) and have more self-evaluative and motivational investment in themselves (Cash, Melnyk & Hrabosky, 2004). Accordingly, females also demonstrated greater concern with disease-related self-care (Hoffman et al., 2021; Sousa et al., 2020). However, attitude to intimate touch (AIT subscale) scores were comparable between female and male participants, which is in line with what was reported by Guerrero & Andersen (1991) in a study with couples, and also with the English and Mongolian TEAQ versions (Trotter et al., 2018a; Tumurbaatar et al., 2022). Finally, males scored higher than females in terms of positive attitude to unfamiliar touch (AUT subscale), which supports previous findings that females avoid and find the touch of strangers more unpleasant than males (Hertenstein et al., 2006).

Irrespective of current findings, some limitations should be considered. First, the data was collected during the outbreak of the COVID-19 pandemic, which can influence the way people perceive interpersonal touch due to the social distancing measures imposed. However, the validity and reliability of the questionnaire do not appear to have been affected by this factor, since the present values are in agreement with those obtained in the original version. Nevertheless, it may be important to reevaluate the questionnaire after the pandemic is over, exploring possible differences in post-pandemic touch experiences. Also, the majority of the participants were female, single, and with no children, which can make it difficult to obtain a representative sample of the population. Additionally, most participants were young adults (average age below 30), which limits generalisation to older people. This limitation may also be related to the fact that the evaluation was conducted online which limits access to the population with the capacity to use computer applications. Such sampling biases should be taken into account since these are variables that can affect the perception of affective touch. In this respect, previous work has shown a positive correlation between age and pleasantness in the affective touch dimension (Sehlstedt et al., 2016), nevertheless Trotter et al. (2018a) reported a lack of effect of age on TEAQ subscale scores; married people have higher scores on the CIT scale of the TEAQ (Trotter et al., 2018b; Tumurbaatar et al., 2022); and, as previously discussed, female have more positive touch experiences and perceive affective touch as more pleasant (Russo, Ottaviani & Spitoni, 2020). In future investigations, it could also be beneficial to conduct a discriminant analysis comparing with clinical groups such as ASD, anorexia nervosa, or chronic pain patients.

## Conclusions

Overall, the European Portuguese version of the TEAQ demonstrated good psychometric properties. This instrument allows the measurement of both touch experiences (*i.e.,* with friends and family, intimate touch, and during childhood) and attitudes towards touch (*i.e.,* related to self-care, intimate touch, and unfamiliar touch). Therefore, this questionnaire will be a new instrument that can increase knowledge about affective touch and could be beneficial for its application in research and clinical settings, facilitating the assessment interactions between attitudes/experiences towards this sensory modality and other psychosocial and neurobiological dimensions in healthy participants, and how they may be altered in clinical populations. It will also allow us to discriminate the role of past and present attitudes/experiences of touch in the normal and pathological development of individuals.

##  Supplemental Information

10.7717/peerj.14960/supp-1 Data S1 Raw Data

10.7717/peerj.14960/supp-2 Supplemental Information 2 Item-total and mean inter-item correlations

10.7717/peerj.14960/supp-3 Supplemental Information 3 Pearson correlations between items and items means and SD

10.7717/peerj.14960/supp-4 Supplemental Information 4 Multi-group confirmatory factor analysis

10.7717/peerj.14960/supp-5 Appendix A European Portuguese version of the Touch Experiences and Attitudes Questionnaire (TEAQ)

## Additional Information and Declarations

### Data Availability

The following information was supplied regarding data availability: The raw data are available in the Supplemental Files.

## References

[ref-1] Arbuckle JL (2013). IBM^®^ SPSS^®^ AmosTM 22 User’s Guide. Chicago: SPSS, Inc.

[ref-2] Benenson JF, Morash D, Petrakos H (1998). Gender differences in emotional closeness between preschool children and their mothers. Sex Roles.

[ref-3] Berghout Austin AM, Braeger TJ (1990). Gendered differences in parents’ encouragement of sibling interaction: Implications for the construction of a personal premise system. First Language.

[ref-4] Bland JM, Altman DG (1997). Statistics notes: Cronbach’s alpha. British Medical Journal.

[ref-5] Boehme M, Van de Wouw M, Bastiaanssen TFS, Olavarría-Ramírez L, Lyons K, Fouhy F, Golubeva AV, Moloney GM, Minuto C, Sandhu KV, Scott KA, Clarke G, Stanton C, Dinan TG, Schellekens H, Cryan JF (2020). Mid-life microbiota crises: middle age is associated with pervasive neuroimmune alterations that are reversed by targeting the gut microbiome. Molecular Psychiatry.

[ref-6] Bond T, Fox CM (2015). Applying the rasch model: fundamental measurement in the human sciences.

[ref-7] Cameron IM, Scott NW, Adler M, Reid IC (2014). A comparison of three methods of assessing differential item functioning (DIF) in the hospital anxiety depression scale: ordinal logistic regression, Rasch analysis and the Mantel chi-square procedure. Quality of Life Research.

[ref-8] Cascio CJ, Lorenzi J, Baranek GT (2016). Self-reported pleasantness ratings and examiner-coded defensiveness in response to touch in children with ASD: effects of stimulus material and bodily location. Journal of Autism and Developmental Disorders.

[ref-9] Cascio CJ, Moore D, McGlone F (2019). Social touch and human development. Developmental Cognitive Neuroscience.

[ref-10] Cash TF, Melnyk SE, Hrabosky JI (2004). The assessment of body image investment: an extensive revision of the appearance schemas inventory. International Journal of Eating Disorders.

[ref-11] Cochrane N (1990). Physical contact experience and depression. Acta Psychiatrica Scandinavica.

[ref-12] Cohen J (1992). Quantitative methods in psychology: a power primer. Psychological Bulletin.

[ref-13] Croy I, Geide H, Paulus M, Weidner K, Olausson H (2016). Affective touch awareness in mental health and disease relates to autistic traits—an explorative neurophysiological investigation. Psychiatry Research.

[ref-14] Croy I, Sehlstedt I, Wasling HB, Ackerley R, Olausson H (2019). Gentle touch perception: from early childhood to adolescence. Developmental Cognitive Neuroscience.

[ref-15] Crucianelli L, Cardi V, Treasure J, Jenkinson PM, Fotopoulou A (2016). The perception of affective touch in anorexia nervosa. Psychiatry Research.

[ref-16] Debrot A, Stellar JE, MacDonald G, Keltner D, Impett EA (2021). Is touch in romantic relationships universally beneficial for psychological well-being? The role of attachment avoidance. Personality and Social Psychology Bulletin.

[ref-17] Duhn L (2010). The importance of touch in the development of attachment. Advances in Neonatal Care.

[ref-18] Ellingsen D-M, Leknes S, Løseth G, Wessberg J, Olausson H (2016). The neurobiology shaping affective touch: expectation, motivation, and meaning in the multisensory context. Frontiers in Psychology.

[ref-19] Fauth B, Decristan J, Decker A-T, Büttner G, Hardy I, Klieme E, Kunter M (2019). The effects of teacher competence on student outcomes in elementary science education: the mediating role of teaching quality. Teaching and Teacher Education.

[ref-20] Feldman R, Singer M, Zagoory O (2010). Touch attenuates infants’ physiological reactivity to stress. Developmental Science.

[ref-21] Foss-Feig JH, Heacock JL, Cascio CJ (2012). Tactile responsiveness patterns and their association with core features in autism spectrum disorders. Research in Autism Spectrum Disorders.

[ref-22] Gordon I, Zagoory-Sharon O, Leckman JF, Feldman R (2010). Oxytocin, cortisol, and triadic family interactions. Physiology & Behavior.

[ref-23] Guerrero LK, Andersen PA (1991). The waxing and waning of relational intimacy: touch as a function of relational stage, gender and touch avoidance. Journal of Social and Personal Relationships.

[ref-24] Hertenstein MJ, Verkamp JM, Kerestes AM, Holmes RM (2006). The communicative functions of touch in humans, nonhuman primates, and rats: a review and synthesis of the empirical research. Genetic, Social, and General Psychology Monographs.

[ref-25] Hilton A, Skrutkowski M (2002). Translating instruments into other languages: development and testing processes. Cancer Nursing.

[ref-26] Hoffman RD, Thielmann A, Buczkowski K, Edirne T, Hoffmann K, Koskela T, Lingner H, Mevsim V, Tekiner S, Zielinski A, Cicurel NHoffman, Petrazzuoli F, Thulesius H, Gerasimovska Kitanovska B, Weltermann B (2021). Gender differences in self-care for common colds by primary care patients: a European multicenter survey on the prevalence and patterns of practices (the COCO study). Journal of Gender Studies.

[ref-27] Hooper D, Coughlan J, Mullen M (2008). Structural equation modelling: guidelines for determining model fit. Electronic Journal of Business Research Methods.

[ref-28] Hyman SL, Levy SE, Myers SM, Kuo DZ, Apkon S, Davidson LF, Ellerbeck KA, Foster JEA, Noritz GH, Leppert MO, Saunders BS, Stille C, Yin L, Weitzman CC, Childers DO, Levine JM, Peralta-Carcelen AM, Poon JK, Bridgemohan C, COUNCIL ON CHILDREN WITH DISABILITIES, SECTION ON DEVELOPMENTAL AND BEHAVIORAL PEDIATRICS (2020). Identification, evaluation, and management of children with autism spectrum disorder. Pediatrics.

[ref-29] Jönsson EH, Bendas J, Weidner K, Wessberg J, Olausson H, Wasling HB, Croy I (2017). The relation between human hair follicle density and touch perception. Scientific Reports.

[ref-30] Kaiser MD, Yang DY-J, Voos AC, Bennett RH, Gordon I, Pretzsch C, Beam D, Keifer C, Eilbott J, McGlone F, Pelphrey KA (2016). Brain mechanisms for processing affective (and nonaffective) touch are atypical in autism. Cerebral Cortex.

[ref-31] Kemp AH, Guastella AJ (2011). The role of oxytocin in human affect: a novel hypothesis. Current Directions in Psychological Science.

[ref-32] Kline RB (2016). Methodology in the social sciences. Principles and practice of structural equation modeling.

[ref-33] Lin SH, Cermak S, Coster WJ, Miller L (2005). The relation between length of institutionalization and sensory integration in children adopted from eastern europe. The American Journal of Occupational Therapy.

[ref-34] Linacre JM (1999). Investigating rating scale category utility. Journal of Outcome Measurement.

[ref-35] Lovakov A, Agadullina ER (2021). Empirically derived guidelines for effect size interpretation in social psychology. European Journal of Social Psychology.

[ref-36] Magalhães E, Salgueira A, Gonzalez A-J, Costa JJ, Costa MJ, Costa P, Lima MPD (2014). NEO-FFI: psychometric properties of a short personality inventory in Portuguese context. Psicologia: Reflexão e Crítica.

[ref-37] Maier A, Gieling C, Heinen-Ludwig L, Stefan V, Schultz J, Güntürkün O, Becker B, Hurlemann R, Scheele D (2020). Association of childhood maltreatment with interpersonal distance and social touch preferences in adulthood. American Journal of Psychiatry.

[ref-38] Maitre NL, Key AP, Chorna OD, Slaughter JC, Matusz PJ, Wallace MT, Murray MM (2017). The dual nature of early-life experience on somatosensory processing in the human infant brain. Current Biology.

[ref-39] Marshall A (2022). Processing and transmission of affective touch in the spinal cord. Current Opinion in Behavioral Sciences.

[ref-40] McCrae RR, Costa PT (1987). Validation of the five-factor model of personality across instruments and observers. Journal of Personality and Social Psychology.

[ref-41] McGlone F, Wessberg J, Olausson H (2014). Discriminative and affective touch: sensing and feeling. Neuron.

[ref-42] Miguel HO, Gonçalves ÓF, Sampaio A (2020). Behavioral response to tactile stimuli relates to brain response to affective touch in 12-month-old infants. Developmental Psychobiology.

[ref-43] Morrison I (2016). ALE meta-analysis reveals dissociable networks for affective and discriminative aspects of touch. Human Brain Mapping.

[ref-44] Morrison I, Löken LS, Olausson H (2010). The skin as a social organ. Experimental Brain Research.

[ref-45] Moszkowski RJ, Stack DM (2007). Infant touching behaviour during mother–infant face-to-face interactions. Infant and Child Development.

[ref-46] Nasser F, Wisenbaker J (2003). A Monte Carlo study investigating the impact of item parceling on measures of fit in confirmatory factor analysis. Educational and Psychological Measurement.

[ref-47] Nordin M (1990). Low-threshold mechanoreceptive and nociceptive units with unmyelinated (C) fibres in the human supraorbital nerve. The Journal of Physiology.

[ref-48] Olausson H, Lamarre Y, Backlund H, Morin C, Wallin BG, Starck G, Ekholm S, Strigo I, Worsley K, Vallbo ÅB, Bushnell MC (2002). Unmyelinated tactile afferents signal touch and project to insular cortex. Nature Neuroscience.

[ref-49] Olausson H, Wessberg J, Morrison I, McGlone F, Vallbo Å (2010). The neurophysiology of unmyelinated tactile afferents. Neuroscience & Biobehavioral Reviews.

[ref-50] Pallant J (2011). SPSS survival manual. A step by step guide data to analysis using SPSS.

[ref-51] Perkeybile AM, Bales KL (2015). Early rearing experience is related to altered aggression and vasopressin production following chronic social isolation in the prairie vole. Behavioural Brain Research.

[ref-52] Pinto-Gouveia J, Castilho P, Galhardo A, Cunha M (2006). Early maladaptive schemas and social phobia. Cognitive Therapy and Research.

[ref-53] Quittkat HL, Hartmann AS, Düsing R, Buhlmann U, Vocks S (2019). Body dissatisfaction, importance of appearance, and body appreciation in men and women over the lifespan. Frontiers in Psychiatry.

[ref-54] Russo V, Ottaviani C, Spitoni GF (2020). Affective touch: a meta-analysis on sex differences. Neuroscience & Biobehavioral Reviews.

[ref-55] Sehlstedt I, Ignell H, Backlund Wasling H, Ackerley R, Olausson H, Croy I (2016). Gentle touch perception across the lifespan. Psychology and Aging.

[ref-56] Shamay-Tsoory SG, Fischer M, Dvash J, Harari H, Perach-Bloom N, Levkovitz Y (2009). Intranasal administration of oxytocin increases envy and schadenfreude (gloating). Biological Psychiatry.

[ref-57] Sousa CN, Marujo P, Teles P, Lira MN, Dias VFF, Novais MELM (2020). Self-care behavior profiles with arteriovenous fistula in hemodialysis patients. Clinical Nursing Research.

[ref-58] Stack DM, Muir DW (1990). Tactile stimulation as a component of social interchange: new interpretations for the still-face effect. British Journal of Developmental Psychology.

[ref-59] Streiner DL (2003). Starting at the beginning: an introduction to coefficient alpha and internal consistency. Journal of Personality Assessment.

[ref-60] Suvilehto JT, Glerean E, Dunbar RIM, Hari R, Nummenmaa L (2015). Topography of social touching depends on emotional bonds between humans. Proceedings of the National Academy of Sciences of the United States of America.

[ref-61] Tabachnick BG, Fidell LS (2013). Using multivariate statistics.

[ref-62] Trotter PD, Belovol E, McGlone F, Varlamov A (2018b). Validation and psychometric properties of the Russian version of the touch experiences and attitudes questionnaire (TEAQ-37 Rus). PLOS ONE.

[ref-63] Trotter PD, McGlone F, Reniers RLEP, Deakin JFW (2018a). Construction and validation of the touch experiences and attitudes questionnaire (TEAQ): a self-report measure to determine attitudes toward and experiences of positive touch. Journal of Nonverbal Behavior.

[ref-64] Tumurbaatar E, Jargalsaikhan O, Tumur-Ochir G, Belovol E (2022). Reliability and validity of the mongolian version of affective touch questionnaire. Neuroscience Research Notes.

[ref-65] Upenieks L, Schafer MH (2022). Keeping in touch: demographic patterns of interpersonal touch in later life. Research on Aging.

[ref-66] Utari GP, Liliawati W, Utama JA (2021). Design and validation of six-tier astronomy diagnostic test instruments with Rasch model analysis. Journal of Physics: Conference Series.

[ref-67] Vallbo A, Olausson H, Wessberg J, Norrsell U (1993). A system of unmyelinated afferents for innocuous mechanoreception in the human skin. Brain Research.

[ref-68] Vieira AI, Ramos AV, Cavalheiro LM, Almeida P, Nogueira D, Reis E, Nunes MV, Castro-Caldas A (2016). Reliability and validity of the European Portuguese version of the social touch questionnaire. Journal of Nonverbal Behavior.

[ref-69] Walker SC, Trotter PD, Swaney WT, Marshall A, Mcglone FP (2017). C-tactile afferents: cutaneous mediators of oxytocin release during affiliative tactile interactions?. Neuropeptides.

[ref-70] Webb A, Peck J (2015). Individual differences in interpersonal touch: on the development, validation, and use of the comfort with interpersonal touch (CIT) scale. Journal of Consumer Psychology.

[ref-71] Wessberg J, Olausson H, Fernström KW, Vallbo ÅB (2003). Receptive field properties of unmyelinated tactile afferents in the human skin. Journal of Neurophysiology.

[ref-72] Wilbarger J, Gunnar M, Schneider M, Pollak S (2010). Sensory processing in internationally adopted, post-institutionalized children: institutionalization and sensory processing. Journal of Child Psychology and Psychiatry.

[ref-73] Wilhelm FH, Kochar AS, Roth WT, Gross JJ (2001). Social anxiety and response to touch: incongruence between self-evaluative and physiological reactions. Biological Psychology.

[ref-74] Wright BD, Linacre JM (1994). Reasonable mean-square fit values. Rasch Measurement Transactions.

[ref-75] Yang C, Nay S, Hoyle RH (2010). Three approaches to using lengthy ordinal scales in structural equation models: parceling, latent scoring, and shortening scales. Applied Psychological Measurement.

