# Supplementary material for: Translation, validity, and reliability of the European Portuguese version of the Touch Experiences and Attitudes Questionnaire
Source: PeerJ. 2023 Apr 3;11:e14960. doi: 10.7717/peerj.14960 (PMC10078461; doi:10.7717/peerj.14960)
Supplement: Supplemental Information 2 [file peerj-11-14960-s002.pdf]

Item-total and mean inter-item correlations

Table 1 shows the item-total correlations for each item of the TEAQ subscales. The mean inter-item is .46 for FFT, .40 for CIT, .53 for ChT, .46 for ASC, .40 for AIT, and .41 for AUT.

Table 1. Item-total correlations for each item of the TEAQ subscales.

Factor and items	Item-total correlation
FFT	
Q4	.45
Q13	.66
Q14	.70
Q16	.63
Q20	.72
Q28	.72
Q36	.70
Q45	.57
Q48	.56
Q51	.71
Q52	.63
CIT	
Q11	.57
Q17	.54
Q18	.45
Q23	.53
Q25	.63
Q27	.43
Q34	.60
Q39	.75
Q42	.77
Q43	.65
Q46	.60
ChT	
Q5	.79
Q6	.71
Q9	.72
Q15	.65
Q21	.67
Q30	.49
Q31	.81
Q33	.68
ASC	
Q2	.62
Q7	.67
Q40	.64
Q49	.44
Q50	.61
AIT	
Q8	.50
Q10	.52
Q12	.66

Q19	.44
Q22	.66
Q24	.53
Q29	.59
Q32	.66
Q38	.57
Q41	.68
Q44	.69
Q47	.65
AUT	
Q1	.38
Q3	.47
Q26	.55
Q35	.69
Q37	.69
