# Supplementary material for: Translation, validity, and reliability of the European Portuguese version of the Touch Experiences and Attitudes Questionnaire
Source: PeerJ. 2023 Apr 3;11:e14960. doi: 10.7717/peerj.14960 (PMC10078461; doi:10.7717/peerj.14960)
Supplement: Supplemental Information 3 [file peerj-11-14960-s003.pdf]

Pearson correlations between items and items means and standard deviations

Table 1 shows items means and standard deviations and Table 2 shows Pearson correlations between items.

Table 1. Items means and standard deviations

Items	Mean	SD	N
TEAQ_1	3,08	1,24	384
TEAQ_2	3,54	1,28	384
TEAQ_3	1,83	1,07	384
TEAQ_4	3,95	1,15	384
TEAQ_5	3,82	1,17	384
TEAQ_6	3,65	1,27	384
TEAQ_7	3,05	1,31	384
TEAQ_8	4,24	0,97	384
TEAQ_9	3,82	1,28	384
TEAQ_10	4,01	1,09	384
TEAQ_11	3,55	1,31	384
TEAQ_12	4,44	0,84	384
TEAQ_13	3,24	1,36	384
TEAQ_14	3,51	1,34	384
TEAQ_15	4,03	1,01	384
TEAQ_16	3,54	1,21	384
TEAQ_17	3,88	1,18	384
TEAQ_18	4,08	1,01	384
TEAQ_19	4,42	0,80	384
TEAQ_20	3,73	1,21	384
TEAQ_21	3,61	1,39	384
TEAQ_22	4,04	0,98	384
TEAQ_23	2,46	1,40	384
TEAQ_24	4,31	0,95	384
TEAQ_25	3,13	1,38	384
TEAQ_26	1,85	1,04	384
TEAQ_27	3,07	1,20	384
TEAQ_28	3,02	1,31	384
TEAQ_29	4,47	0,77	384
TEAQ_30	4,17	1,05	384
TEAQ_31	3,89	1,15	384
TEAQ_32	4,43	0,85	384
TEAQ_33	4,29	0,98	384
TEAQ_34	3,58	1,39	384
TEAQ_35	2,38	1,17	384
TEAQ_36	3,73	1,01	384
TEAQ_37	2,24	1,13	384
TEAQ_38	4,15	0,94	384
TEAQ_39	3,49	1,42	384
TEAQ_40	3,52	1,25	384
TEAQ_41	4,59	0,68	384
TEAQ_42	3,29	1,28	384
TEAQ_43	3,94	1,11	384
TEAQ_44	4,34	0,89	384

TEAQ_45	3,12	1,25	384
TEAQ_46	3,07	1,45	384
TEAQ_47	4,18	0,91	384
TEAQ_48	2,49	1,27	384
TEAQ_49	2,95	1,32	384
TEAQ_50	3,27	1,47	384
TEAQ_51	3,86	1,11	384
TEAQ_52	2,80	1,38	384

Table 2. Pearson correlations between items.

		Q1	Q2	Q3	Q4	Q5	Q6	Q7	Q8	Q9	Q10	Q11	Q12	Q13	Q14	Q15	Q16	Q17	Q18	Q19	Q20	Q21	Q22	Q23	Q24	Q25	Q26	Q27	Q28	Q29	Q30	Q31	Q32	Q33	Q34	Q35	Q36	Q37	Q38	Q39	Q40	Q41	Q42	Q43	Q44	Q45	Q46	Q47	Q48	Q49	Q50	Q51	Q52
**Q1**	r	1	.153	.242	.213	.150	.232	0.082	.227	.168	.169	0.082	.179	.314	.376	.244	.312	.131	.184	.175	.489	.140	.259	0.001	.181	-0.041	.311	.184	.376	.343	.120	.217	.179	.126	.165	.315	.393	.314	.240	-0.011	0.075	.128	0.054	.111	.171	.352	0.072	.274	.296	0.042	0.034	.447	.197
	p		0.003	<.001	<.001	0.003	<.001	0.108	<.001	<.001	<.001	0.11	<.001	<.001	<.001	<.001	<.001	0.01	<.001	<.001	<.001	0.006	<.001	0.981	<.001	0.422	<.001	<.001	<.001	<.001	0.019	<.001	<.001	0.013	0.001	<.001	<.001	<.001	<.001	0.836	0.141	0.012	0.287	0.03	<.001	<.001	0.157	<.001	<.001	0.412	0.509	<.001	<.001
**Q2**	r		1	-0.007	.244	0.095	.140	.608	.219	0.069	.235	.211	.184	.254	.135	.217	.334	.320	.215	.138	.227	.128	.254	.213	.200	.167	0	.145	.199	.243	.209	.136	.225	.179	.188	0.067	.166	0.055	.254	.171	.502	.217	.283	.260	.201	.190	.194	.294	.192	.305	.481	.188	.255
	p			0.89	<.001	0.063	0.006	<.001	<.001	0.176	<.001	<.001	<.001	<.001	0.008	<.001	<.001	<.001	<.001	0.007	<.001	0.012	<.001	<.001	<.001	0.001	0.996	0.005	<.001	<.001	<.001	0.007	<.001	<.001	<.001	0.191	0.001	0.286	<.001	<.001	<.001	<.001	<.001	<.001	<.001	<.001	<.001	<.001	<.001	<.001	<.001	<.001	<.001
**Q3**	r			1	0.067	0.08	.136	0.047	0.003	0.083	0.056	0.078	0.01	.156	.177	0.022	.145	0.016	0.051	-0.054	.180	-0.034	.119	0.066	0.03	0.076	.391	0.074	.197	0.007	-0.069	0.008	0.049	-0.019	.117	.405	.154	.407	0.031	-0.016	-0.031	-0.07	0.067	0.084	0.046	.218	0.014	0.027	.178	0.014	-0.065	.145	0.09
	p				0.192	0.115	0.008	0.355	0.952	0.104	0.276	0.126	0.843	0.002	<.001	0.668	0.005	0.758	0.321	0.293	<.001	0.506	0.02	0.198	0.553	0.14	<.001	0.146	<.001	0.891	0.179	0.874	0.343	0.718	0.021	<.001	0.002	<.001	0.545	0.758	0.544	0.172	0.189	0.102	0.372	<.001	0.783	0.598	<.001	0.777	0.206	0.004	0.078
**Q4**	r				1	.233	.303	.196	.225	.121	.238	.212	.192	.228	.312	.271	.626	.185	.208	.123	.362	.159	.180	0.096	.152	0.085	0.02	.201	.355	.320	.181	.251	.212	.275	.344	0.061	.365	.124	.244	.158	.143	.232	.215	.244	.162	.281	0.063	.269	.176	.131	.144	.438	.231
	p					<.001	<.001	<.001	<.001	0.018	<.001	<.001	<.001	<.001	<.001	<.001	<.001	<.001	<.001	0.016	<.001	0.002	<.001	0.06	0.003	0.098	0.699	<.001	<.001	<.001	<.001	<.001	<.001	<.001	<.001	0.233	<.001	0.015	<.001	0.002	0.005	<.001	<.001	<.001	0.001	<.001	0.217	<.001	<.001	0.01	0.005	<.001	<.001
**Q5**	r					1	.729	.108	.116	.729	.127	.123	0.065	.276	.330	.536	.319	.231	.270	.172	.268	.543	.173	.122	0.076	.172	0.083	.262	.320	.188	.381	.679	.130	.577	.365	.156	.353	.159	.130	.138	0.007	.172	.242	.218	0.075	.318	.137	.108	.143	0.096	0.074	.295	.225
	p						<.001	0.034	0.023	<.001	0.013	0.016	0.206	<.001	<.001	<.001	<.001	<.001	<.001	<.001	<.001	<.001	<.001	0.016	0.139	<.001	0.105	<.001	<.001	<.001	<.001	<.001	0.011	<.001	<.001	0.002	<.001	0.002	0.011	0.007	0.884	<.001	<.001	<.001	0.141	<.001	0.007	0.035	0.005	0.061	0.146	<.001	<.001
**Q6**	r						1	0.09	0.076	.669	0.053	.144	.103	.380	.452	.538	.388	.236	.290	.191	.431	.474	.163	.101	.130	.153	0.088	.233	.437	.241	.296	.586	.152	.500	.349	.129	.444	.160	.199	.149	0.028	.157	.235	.271	.105	.365	.146	.175	.236	0.068	0.052	.399	.334
	p							0.079	0.139	<.001	0.303	0.005	0.043	<.001	<.001	<.001	<.001	<.001	<.001	<.001	<.001	<.001	0.001	0.048	0.011	0.003	0.085	<.001	<.001	<.001	<.001	<.001	0.003	<.001	<.001	0.011	<.001	0.002	<.001	0.003	0.58	0.002	<.001	<.001	0.04	<.001	0.004	<.001	<.001	0.182	0.311	<.001	<.001
**Q7**	r							1	.241	0.077	.206	.222	.150	.261	.136	.176	.256	.264	.206	0.093	.149	.207	.208	.211	.115	.150	0.011	.144	.219	.184	.205	.164	.183	.140	.203	0.048	.141	0.029	.197	.185	.533	.242	.301	.261	.240	.176	.159	.179	.203	.435	.454	.126	.268
	p								<.001	0.131	<.001	<.001	0.003	<.001	0.007	<.001	<.001	<.001	<.001	0.069	0.003	<.001	<.001	<.001	0.024	0.003	0.823	0.005	<.001	<.001	<.001	0.001	<.001	0.006	<.001	0.346	0.006	0.576	<.001	<.001	<.001	<.001	<.001	<.001	<.001	<.001	0.002	<.001	<.001	<.001	<.001	0.014	<.001
**Q8**	r								1	0.087	.384	.329	.342	.221	.186	.198	.238	.267	.174	.187	.267	.135	.423	.177	.263	.101	-0.001	.132	.149	.400	.219	.208	.348	.162	.221	0.062	.203	0.052	.336	.215	.176	.364	.212	.260	.356	.138	.143	.352	.161	.125	.178	.234	.213
	p									0.089	<.001	<.001	<.001	<.001	<.001	<.001	<.001	<.001	<.001	<.001	<.001	0.008	<.001	<.001	<.001	0.049	0.992	0.01	0.004	<.001	<.001	<.001	<.001	0.001	<.001	0.227	<.001	0.309	<.001	<.001	<.001	<.001	<.001	<.001	<.001	0.007	0.005	<.001	0.002	0.014	<.001	<.001	<.001
**Q9**	r									1	0.034	.113	0.032	.230	.283	.461	.180	.150	.229	0.063	.223	.543	.116	0.01	0.041	.101	.139	.206	.288	.127	.277	.656	0.045	.477	.335	0.099	.360	.103	0.058	0.097	0.004	.116	.155	.110	0.037	.287	0.088	0.094	.186	0.064	0.071	.233	.221
	p										0.511	0.027	0.529	<.001	<.001	<.001	<.001	0.003	<.001	0.216	<.001	<.001	0.023	0.852	0.423	0.048	0.006	<.001	<.001	0.013	<.001	<.001	0.382	<.001	<.001	0.053	<.001	0.043	0.26	0.057	0.945	0.023	0.002	0.032	0.473	<.001	0.084	0.066	<.001	0.209	0.167	<.001	<.001
**Q10**	r										1	.520	.364	.231	.119	.181	.248	.331	.173	.194	.293	.119	.395	.330	.276	.247	0.034	.176	.139	.294	.154	.111	.354	.141	.308	0.021	0.098	0.046	.386	.318	.185	.323	.332	.307	.435	.116	.480	.493	.160	.220	.172	.167	.171
	p											<.001	<.001	<.001	0.02	<.001	<.001	<.001	<.001	<.001	<.001	0.019	<.001	<.001	<.001	<.001	0.504	<.001	0.006	<.001	0.002	0.029	<.001	0.006	<.001	0.686	0.055	0.364	<.001	<.001	<.001	<.001	<.001	<.001	<.001	0.023	<.001	<.001	0.002	<.001	<.001	0.001	<.001
**Q11**	r											1	.343	.287	.210	.159	.285	.368	.239	.140	.336	0.068	.312	.406	.286	.355	0.043	.224	.205	.243	.127	.115	.254	.198	.411	-0.041	.216	-0.046	.320	.474	0.08	.299	.462	.452	.328	.198	.468	.524	.259	.193	.128	.150	.258
	p												<.001	<.001	<.001	0.002	<.001	<.001	<.001	0.006	<.001	0.182	<.001	<.001	<.001	<.001	0.397	<.001	<.001	<.001	0.013	0.024	<.001	<.001	<.001	0.424	<.001	0.372	<.001	<.001	0.118	<.001	<.001	<.001	<.001	<.001	<.001	<.001	<.001	<.001	0.012	0.003	<.001
**Q12**	r												1	.126	.165	.148	.258	.351	.137	.379	.268	0.064	.489	.296	.665	.332	.124	.108	.104	.327	.182	0.068	.472	.142	.231	.120	.175	.112	.301	.419	.161	.614	.270	.346	.454	.157	.222	.463	0.083	0.063	0.08	.156	0.055
	p													0.013	0.001	0.004	<.001	<.001	0.007	<.001	<.001	0.211	<.001	<.001	<.001	<.001	0.015	0.034	0.041	<.001	<.001	0.186	<.001	0.005	<.001	0.019	<.001	0.028	<.001	<.001	0.002	<.001	<.001	<.001	<.001	0.002	<.001	<.001	0.106	0.214	0.118	0.002	0.279
**Q13**	r													1	.508	.307	.421	.325	.234	.143	.513	.199	.272	.163	0.065	0.076	.146	.163	.481	.307	.211	.234	.195	.309	.327	.109	.492	0.075	.294	.152	.171	.180	.243	.313	.232	.345	.209	.287	.495	.154	.162	.498	.698
	p														<.001	<.001	<.001	<.001	<.001	0.005	<.001	<.001	<.001	0.001	0.201	0.138	0.004	0.001	<.001	<.001	<.001	<.001	<.001	<.001	<.001	0.032	<.001	0.145	<.001	0.003	<.001	<.001	<.001	<.001	<.001	<.001	<.001	<.001	<.001	0.002	0.001	<.001	<.001
**Q14**	r														1	.407	.461	.199	.207	.195	.602	.270	.311	0.08	.137	0.046	.151	.172	.692	.439	.245	.368	.245	.327	.269	.194	.566	.164	.280	.136	0.085	.264	.215	.287	.249	.457	.127	.288	.374	.112	0.048	.592	.415
	p															<.001	<.001	<.001	<.001	<.001	<.001	<.001	<.001	0.119	0.007	0.365	0.003	<.001	<.001	<.001	<.001	<.001	<.001	<.001	<.001	<.001	<.001	0.001	<.001	0.008	0.097	<.001	<.001	<.001	<.001	<.001	0.013	<.001	<.001	0.028	0.346	<.001	<.001
**Q15**	r															1	.447	.225	.318	.135	.367	.466	.242	0.047	.135	.109	0.079	.229	.392	.332	.411	.624	.188	.523	.326	.111	.379	.101	.193	.100	.163	.219	.192	.255	.160	.317	.151	.231	.192	.158	.147	.360	.311
	p																<.001	<.001	<.001	0.008	<.001	<.001	<.001	0.356	0.008	0.032	0.12	<.001	<.001	<.001	<.001	<.001	<.001	<.001	<.001	0.029	<.001	0.047	<.001	0.05	0.001	<.001	<.001	<.001	0.002	<.001	0.003	<.001	<.001	0.002	0.004	<.001	<.001
**Q16**	r																1	.381	.327	.225	.507	.259	.348	.194	.214	.138	.118	.287	.472	.392	.287	.332	.225	.337	.391	.143	.478	.156	.331	.224	.245	.283	.314	.386	.242	.391	.142	.351	.310	.177	.182	.544	.352
	p																	<.001	<.001	<.001	<.001	<.001	<.001	<.001	<.001	0.007	0.021	<.001	<.001	<.001	<.001	<.001	<.001	<.001	<.001	0.005	<.001	0.002	<.001	<.001	<.001	<.001	<.001	<.001	<.001	<.001	0.005	<.001	<.001	<.001	<.001	<.001	<.001
**Q17**	r																	1	.293	.226	.335	.187	.366	.288	.306	.323	-0.007	.227	.214	.298	.165	.193	.388	.270	.399	0.017	.225	0.001	.541	.451	.213	.425	.424	.652	.413	0.088	.301	.416	.180	.215	.240	.219	.268
	p																		<.001	<.001	<.001	<.001	<.001	<.001	<.001	<.001	0.896	<.001	<.001	<.001	0.001	<.001	<.001	<.001	<.001	0.737	<.001	0.985	<.001	<.001	<.001	<.001	<.001	<.001	<.001	0.087	<.001	<.001	<.001	<.001	<.001	<.001	<.001
**Q18**	r																		1	.179	.355	.178	.163	.212	.101	.219	0.018	.528	.223	.222	.270	.354	0.071	.201	.361	0.044	.314	0.067	.222	.301	.171	.200	.372	.336	.169	.189	.264	.265	.197	.179	.230	.243	.227
	p																			<.001	<.001	<.001	0.001	<.001	0.047	<.001	0.722	<.001	<.001	<.001	<.001	<.001	0.164	<.001	<.001	0.389	<.001	0.192	<.001	<.001	<.001	<.001	<.001	<.001	<.001	<.001	<.001	<.001	<.001	<.001	<.001	<.001	<.001
**Q19**	r																			1	.247	.145	.311	.153	.308	0.097	.101	.282	.168	.350	.146	.150	.312	.145	.147	0.099	.199	0.073	.259	.225	.128	.464	.139	.196	.292	.187	.140	.336	0.069	.107	0.07	.157	0.085
	p																				<.001	0.005	<.001	0.003	<.001	0.057	0.049	<.001	<.001	<.001	0.004	0.003	<.001	0.005	0.004	0.052	<.001	0.156	<.001	<.001	0.012	<.001	0.006	<.001	<.001	<.001	0.006	<.001	0.175	0.036	0.173	0.002	0.096
**Q20**	r																				1	.175	.393	.182	.228	.107	.165	.274	.609	.501	.214	.266	.312	.248	.423	.189	.586	.212	.410	.267	.146	.294	.336	.406	.351	.478	.264	.425	.449	.128	0.078	.565	.466
	p																					<.001	<.001	<.001	<.001	0.036	0.001	<.001	<.001	<.001	<.001	<.001	<.001	<.001	<.001	<.001	<.001	<.001	<.001	<.001	0.004	<.001	<.001	<.001	<.001	<.001	<.001	<.001	<.001	0.012	0.125	<.001	<.001
**Q21**	r																					1	0.09	0.03	0.004	0.056	-0.05	.173	.252	.127	.423	.651	0.089	.539	.294	0.006	.283	-0.016	.113	0.063	.161	.113	.114	0.095	0.024	.181	0.078	.146	.152	0.093	0.097	.230	.181
	p																						0.077	0.558	0.944	0.275	0.33	<.001	<.001	0.012	<.001	<.001	0.081	<.001	<.001	0.9	<.001	0.748	0.027	0.215	0.002	0.027	0.026	0.062	0.646	<.001	0.128	0.004	0.003	0.07	0.058	<.001	<.001
**Q22**	r																						1	.260	.360	.147	.140	.196	.272	.455	.142	.115	.547	.187	.248	.144	.327	.131	.445	.262	.171	.426	.309	.395	.575	.250	.238	.469	.163	0.088	.103	.307	.223
	p																							<.001	<.001	0.004	0.006	<.001	<.001	<.001	0.005	0.025	<.001	<.001	<.001	0.005	<.001	0.01	<.001	<.001	<.001	<.001	<.001	<.001	<.001	<.001	<.001	<.001	0.001	0.084	0.043	<.001	<.001
**Q23**	r																							1	.331	.511	.109	.245	.105	0.053	0.055	0.073	.210	0.026	.230	0.05	0.046	0.01	.169	.500	0.097	.220	.425	.321	.263	.151	.424	.263	.115	.188	0.094	0.011	.102
	p																								<.001	<.001	0.033	<.001	0.04	0.3	0.283	0.156	<.001	0.611	<.001	0.327	0.373	0.85	<.001	<.001	0.057	<.001	<.001	<.001	<.001	0.003	<.001	<.001	0.024	<.001	0.067	0.835	0.046
**Q24**	r																								1	.467	.143	0.092	0.096	.270	.141	0.073	.462	.125	.242	0.059	.118	0.055	.210	.419	.128	.497	.298	.263	.365	.150	.239	.346	0.002	0.028	0.049	.136	-0.009
	p																									<.001	0.005	0.072	0.059	<.001	0.005	0.151	<.001	0.015	<.001	0.249	0.021	0.281	<.001	<.001	0.012	<.001	<.001	<.001	<.001	0.003	<.001	<.001	0.964	0.579	0.339	0.008	0.862
**Q25**	r																									1	0.03	.256	0.059	-0.026	0.051	.104	.155	0.071	.419	-0.028	-0.021	-0.057	0.093	.666	.109	.215	.625	.400	.203	0.071	.414	.206	0.07	.103	.113	-0.002	0.056
	p																										0.555	<.001	0.249	0.608	0.32	0.042	0.002	0.163	<.001	0.588	0.684	0.263	0.068	<.001	0.033	<.001	<.001	<.001	<.001	0.166	<.001	<.001	0.171	0.043	0.027	0.974	0.271
**Q26**	r																										1	.124	.215	0.083	-0.044	0.061	0.082	0.025	0.069	.478	.173	.463	-0.009	-0.034	-0.055	-0.002	0.024	-0.007	0.049	.315	0.092	0.048	.256	-0.041	-.150	.171	.108
	p																											0.015	<.001	0.103	0.391	0.233	0.11	0.629	0.176	<.001	<.001	<.001	0.853	0.509	0.284	0.974	0.634	0.885	0.339	<.001	0.072	0.348	<.001	0.419	0.003	<.001	0.035
**Q27**	r																											1	.245	.194	.220	.303	.137	.145	.343	.178	.248	.189	.165	.284	0.091	.155	.375	.289	.130	.280	.265	.215	.193	.160	.151	.184	.160
	p																												<.001	<.001	<.001	<.001	0.007	0.004	<.001	<.001	<.001	<.001	0.001	<.001	0.074	0.002	<.001	<.001	0.011	<.001	<.001	<.001	<.001	0.002	0.003	<.001	0.002
**Q28**	r																												1	.402	.195	.327	.214	.291	.325	.197	.562	.205	.256	.117	.155	.203	.244	.251	.202	.487	.204	.212	.422	.125	0.091	.586	.469
	p																													<.001	<.001	<.001	<.001	<.001	<.001	<.001	<.001	<.001	<.001	0.022	0.002	<.001	<.001	<.001	<.001	<.001	<.001	<.001	<.001	0.014	0.076	<.001	<.001
**Q29**	r																													1	.246	.214	.481	.308	.231	.145	.435	.144	.493	.124	.163	.472	.184	.334	.441	.320	.145	.435	.193	.139	.110	.478	.293
	p																														<.001	<.001	<.001	<.001	<.001	0.004	<.001	0.005	<.001	0.015	0.001	<.001	<.001	<.001	<.001	<.001	0.004	<.001	<.001	0.006	0.031	<.001	<.001
**Q30**	r																														1	.485	.154	.483	.240	0.043	.281	-0.002	.139	.124	.221	.172	.151	.179	.153	.244	0.072	.224	0.092	.144	.201	.278	.162
	p																															<.001	0.003	<.001	<.001	0.404	<.001	0.972	0.006	0.015	<.001	<.001	0.003	<.001	0.003	<.001	0.16	<.001	0.071	0.005	<.001	<.001	0.001
**Q31**	r																															1	0.095	.624	.361	0.089	.378	0.058	.161	.103	.146	.136	.185	.186	0.068	.306	0.078	.146	.173	.118	.148	.312	.230
	p																																0.064	<.001	<.001	0.08	<.001	0.258	0.002	0.043	0.004	0.008	<.001	<.001	0.182	<.001	0.129	0.004	<.001	0.021	0.004	<.001	<.001
**Q32**	r																																1	.230	.235	.110	.239	.137	.435	.264	0.084	.494	.253	.362	.587	.131	.254	.426	0.09	0.041	-0.009	.258	.133
	p																																	<.001	<.001	0.031	<.001	0.007	<.001	<.001	0.102	<.001	<.001	<.001	<.001	0.01	<.001	<.001	0.079	0.421	0.854	<.001	0.009
**Q33**	r																																	1	.310	0.006	.353	-0.007	.208	.124	.128	.191	.135	.223	.135	.216	0.053	.211	0.089	0.077	.112	.345	.304
	p																																		<.001	0.905	<.001	0.896	<.001	0.015	0.012	<.001	0.008	<.001	0.008	<.001	0.303	<.001	0.082	0.132	0.028	<.001	<.001
**Q34**	r																																		1	0.064	.330	0.083	.248	.490	.149	.238	.614	.442	.220	.201	.404	.325	.231	0.093	0.09	.243	.277
	p																																			0.213	<.001	0.106	<.001	<.001	0.003	<.001	<.001	<.001	<.001	<.001	<.001	<.001	<.001	0.069	0.079	<.001	<.001
**Q35**	r																																			1	.152	.804	-0.011	-0.058	-0.018	0.074	0.076	0.025	0.065	.326	-0.004	0.086	.211	-0.029	-0.056	.250	.119
	p																																				0.003	<.001	0.835	0.259	0.731	0.149	0.139	0.631	0.206	<.001	0.942	0.093	<.001	0.572	0.276	<.001	0.02
**Q36**	r																																				1	.185	.243	0.092	.112	.269	.192	.267	.221	.453	.114	.298	.432	0.069	.128	.576	.470
	p																																					<.001	<.001	0.071	0.028	<.001	<.001	<.001	<.001	<.001	0.026	<.001	<.001	0.18	0.012	<.001	<.001
**Q37**	r																																					1	-0.014	-0.062	-0.024	0.064	0.047	0.011	0.062	.289	0.022	0.073	.196	-0.006	-0.031	.245	.120
	p																																						0.785	0.226	0.633	0.211	0.363	0.83	0.225	<.001	0.661	0.154	<.001	0.899	0.545	<.001	0.019
**Q38**	r																																						1	.307	.183	.404	.241	.602	.510	.200	.248	.483	.265	.186	.133	.311	.319
	p																																							<.001	<.001	<.001	<.001	<.001	<.001	<.001	<.001	<.001	<.001	<.001	0.009	<.001	<.001
**Q39**	r																																							1	.108	.344	.675	.563	.406	.151	.530	.375	.144	.150	.134	0.045	.107
	p																																								0.034	<.001	<.001	<.001	<.001	0.003	<.001	<.001	0.005	0.003	0.009	0.382	0.036
**Q40**	r																																								1	.231	.200	.202	.129	0.092	.105	.170	.110	.322	.575	.124	.196
	p																																									<.001	<.001	<.001	0.011	0.07	0.039	<.001	0.03	<.001	<.001	0.015	<.001
**Q41**	r																																									1	.270	.401	.517	.139	.201	.465	0.09	.161	.177	.263	.152
	p																																										<.001	<.001	<.001	0.006	<.001	<.001	0.078	0.002	<.001	<.001	0.003
**Q42**	r																																										1	.537	.378	.178	.549	.323	.204	.234	.216	.143	.227
	p																																											<.001	<.001	<.001	<.001	<.001	<.001	<.001	<.001	0.005	<.001
**Q43**	r																																											1	.504	.196	.404	.484	.258	.222	.154	.208	.306
	p																																												<.001	<.001	<.001	<.001	<.001	<.001	0.002	<.001	<.001
**Q44**	r																																												1	.215	.357	.549	.145	.155	0.068	.217	.178
	p																																													<.001	<.001	<.001	0.005	0.002	0.184	<.001	<.001
**Q45**	r																																													1	.173	.280	.435	.118	.113	.462	.356
	p																																														<.001	<.001	<.001	0.02	0.027	<.001	<.001
**Q46**	r																																														1	.365	.269	.152	0.055	0.071	.207
	p																																															<.001	<.001	0.003	0.278	0.166	<.001
**Q47**	r																																															1	.235	.164	.142	.312	.211
	p																																																<.001	0.001	0.005	<.001	<.001
**Q48**	r																																																1	.155	.119	.344	.559
	p																																																	0.002	0.02	<.001	<.001
**Q49**	r																																																	1	.351	.117	.176
	p																																																		<.001	0.022	<.001
**Q50**	r																																																		1	0.093	.225
	p																																																			0.067	<.001
**Q51**	r																																																			1	.444
	p																																																				<.001
**Q52**	r																																																				1
	p																																																				
