# Supplementary material for: Translation, validity, and reliability of the European Portuguese version of the Touch Experiences and Attitudes Questionnaire
Source: PeerJ. 2023 Apr 3;11:e14960. doi: 10.7717/peerj.14960 (PMC10078461; doi:10.7717/peerj.14960)
Supplement: Supplemental Information 4 [file peerj-11-14960-s004.pdf]

Multi-group Confirmatory Factor Analysis

A multi-group confirmatory factor analysis between the two groups (female and male) was made to examine factor invariance. Tables 1 and 2 provide the factors, items, and standardized loadings for the combined, female, and male data, for the six-factor model and for the parcelled six-factor model, respectively. These results show that the items are comparable separate and combined, functioning in a similar way across groups, and that both can produce equivalent results.

Table 1. Standardized loadings for the six-factor model for combined, female, and male data.

	**Factor loadings**		
**Factor and items**	**Combined data**	**Female**	**Male**
FFT			
Q4	.59	.60	.54
Q13	.68	.66	.73
Q14	.76	.75	.82
Q16	.67	.69	.59
Q20	.78	.77	.80
Q28	.77	.76	.81
Q36	.74	.74	.75
Q45	.61	.61	.64
Q48	.57	.55	.61
Q51	.70	.71	.66
Q52	.64	.62	.68
CIT			
Q11	.61	.60	.57
Q17	.61	.61	.55
Q18	.45	.37	.67
Q23	.56	.64	.27
Q25	.68	.75	.52
Q27	.44	.37	.62
Q34	.66	.62	.74
Q39	.81	.83	.78
Q42	.82	.84	.69
Q43	.71	.69	.69
Q46	.64	.64	.62
ChT			
Q5	.84	.86	.82
Q6	.78	.77	.84
Q9	.78	.76	.89
Q15	.69	.74	.53
Q21	.69	.69	.72
Q30	.51	.52	.41
Q31	.84	.85	.74
Q33	.71	.72	.64
ASC			
Q2	.74	.69	.78
Q7	.77	.75	.80
Q40	.71	.66	.58
Q49	.50	.45	.41

Q50	.66	.57	.50
AIT			
Q8	.53	.50	.60
Q10	.56	.62	.40
Q12	.69	.70	.72
Q19	.47	.50	.41
Q22	.71	.70	.70
Q24	.58	.62	.61
Q29	.63	.58	.72
Q32	.70	.70	.77
Q38	.62	.63	.57
Q41	.71	.69	.81
Q44	.75	.73	.80
Q47	.70	.69	.71
AUT			
Q1	.39	.34	.59
Q3	.48	.42	.58
Q26	.55	.54	.52
Q35	.89	.91	.80
Q37	.89	.89	.84

Table 2. Standardized loadings for the parcelled six-factor model for combined, female, and male data.

	**Factor loadings**		
**Factor and items**	**Combined data**	**Female**	**Male**
FFT			
Q28, 48, 52	.81	.80	.83
Q13, 14, 20, 51	.93	.92	.96
Q4, 16, 36, 45	.80	.79	.79
CIT			
Q17, 18, 34, 39	.89	.89	.98
Q11, 23, 25, 27	.79	.82	.64
Q42, 43, 46	.88	.87	.80
ChT			
Q9, 21	.81	.81	.81
Q5, 15, 30	.86	.86	.86
Q6, 31, 33	.97	.97	.92
ASC			
Q50	.68	.58	.54
Q2, 7	.78	.75	.82
Q40, 49	.79	.73	.70
AIT			
Q19, 24, 32, 44	.83	.85	.86
Q8, 12, 41	.81	.81	.83
Q10, 22, 29, 38, 47	.86	.85	.84
AUT			
Q37	.74	.73	.72
Q1, 35	.93	.92	.94
Q3, 26	.59	.55	.65
