# Supplementary material for: Translation, validity, and reliability of the European Portuguese version of the Touch Experiences and Attitudes Questionnaire
Source: PeerJ. 2023 Apr 3;11:e14960. doi: 10.7717/peerj.14960 (PMC10078461; doi:10.7717/peerj.14960)
Supplement: Appendix A [file peerj-11-14960-s005.pdf]

European Portuguese version of the Touch Experiences and Attitudes Questionnaire (TEAQ)

Por favor, leia com atenção as seguintes afirmações e selecione a opção que indica o quanto concorda ou discorda com cada uma das afirmações.

	Discordo completamente	Discordo um pouco	Não concordo nem discordo	Concordo um pouco	Concordo completamente
1. Eu não gosto quando as pessoas são muito afetuosas fisicamente em relação a mim.					
2. Eu gosto de usar loções corporais.					
3. Eu tenho de conhecer alguém relativamente bem para desfrutar do seu abraço.					
4. Eu considero que é natural cumprimentar os meus amigos e familiares com um beijo na bochecha.					
5. Houve muito afeto físico durante a minha infância.					
6. Em criança, eu costumava abraçar os membros da minha família frequentemente.					
7. Eu gosto de usar essências de banho quando tomo banho.					
8. Eu considero que acariciar o cabelo de uma pessoa que gosto é muito agradável.					
9. Os meus pais não foram muito afetuosos fisicamente em relação a mim durante a minha infância.					
10. Eu gosto de adormecer nos braços de alguém de quem sou próximo(a).					
11. Eu costumo aconchegar-me no sofá com alguém.					
12. Eu gosto da intimidade física dos preliminares sexuais.					

	Discordo completamente	Discordo um pouco	Não concordo nem discordo	Concordo um pouco	Concordo completamente
13. Eu gosto de dar o braço aos meus amigos e familiares enquanto ando.					
14. Eu normalmente abraço os meus familiares e amigos quando estou a despedir-me.					
15. Em criança, receber um abraço dos meus pais quando eu estava triste fazia com que eu me sentisse muito mais feliz.					
16. É bom quando amigos e membros da família me cumprimentam com um beijo.					
17. Eu costumo dar as mãos a alguém que conheço intimamente.					
18. Quando eu estou triste, geralmente há alguém que me consegue confortar.					
19. Beijar é uma boa forma de expressar atração física.					
20. Eu abraço regularmente pessoas de quem sou próximo(a).					
21. Em criança, os meus pais aconchegavam-me na cama todas as noites e davam-me um abraço e um beijo de boa noite.					
22. Eu gosto quando a minha pele é acariciada.					
23. Eu costumo tomar um duche ou um banho com alguém.					
24. Eu gosto de fazer sexo.					
25. Eu faço sexo frequentemente.					
26. Eu não gosto de ter proximidade física com pessoas que não conheço bem.					

	Discordo completamente	Discordo um pouco	Não concordo nem discordo	Concordo um pouco	Concordo completamente
27. Eu consigo sempre encontrar alguém para me confortar fisicamente quando não me sinto bem					
28. Eu cumprimento sempre os meus amigos e familiares dando-lhes um abraço.					
29. Eu aprecio ser abraçado(a) por alguém de quem gosto.					
30. A minha mãe dava-me banho regularmente quando eu era criança.					
31. Em criança, os meus pais confortavam-me sempre quando eu estava triste.					
32. Eu gosto da sensação da minha pele a tocar na de outra pessoa se eu a conhecer intimamente.					
33. Em criança, os meus pais costumavam dar-me a mão quando eu caminhava com eles.					
34. Na maioria dos dias eu recebo um abraço ou um beijo.					
35. Se alguém que eu não conheço muito bem coloca a sua mão de forma amigável no meu braço, isso faz-me sentir desconfortável.					
36. Eu costumo ter contacto físico com os meus amigos e familiares quando estou com eles.					
37. Faz-me sentir desconfortável se alguém que eu não conheço muito bem me toca de uma maneira amigável.					
38. Eu aprecio dar a mão a alguém de quem gosto.					
39. Eu costumo partilhar um beijo romântico com frequência.					
40. Eu gosto de exfoliar a minha pele.					

	Discordo completamente	Discordo um pouco	Não concordo nem discordo	Concordo um pouco	Concordo completamente
41. Beijar é uma parte agradável de expressar sentimentos românticos.					
42. A minha pele é acariciada frequentemente.					
43. Eu costumo dar a mão a alguém de quem gosto.					
44. Eu gosto de acariciar a pele de alguém que eu conheço intimamente.					
45. Eu consigo ser abraçado(a) por relativamente muitas pessoas.					
46. Eu costumo adormecer enquanto abraço alguém de quem eu sou próximo(a).					
47. Abraçar alguém no sofá é muito bom.					
48. Eu costumo colocar o braço sobre um amigo próximo enquanto caminhamos juntos.					
49. Eu gosto de tomar banho com muita espuma.					
50. Eu gosto de usar máscaras faciais na minha pele.					
51. Eu gosto quando os meus amigos e familiares me cumprimentam dando-me um abraço.					
52. Eu costumo andar de braços dados com os meus amigos e familiares quando passeamos.					

The item numbers below identify which subscale each item corresponds to (**R** identifies items that are reverse scored):

Friends and family touch (FFT) (11 items): 4, 13, 14, 16, 20, 28, 36, 45, 48, 51, 52;

Current intimate touch (CIT) (11 items): 11, 17, 18, 23, 25, 27, 34, 39, 42, 43, 46;

Childhood touch (ChT) (8 items): 5, 6, 9**R**, 15, 21, 30, 31, 33;

Attitude to self-care (ASC) (5 items): 2, 7, 40, 49, 50;

Attitude to intimate touch (AIT) (12 items): 8, 10, 12, 19, 22, 24, 29, 32, 38, 41, 44, 47;

Attitude to unfamiliar touch (AUT) (5 items): 1**R,** 3**R**, 26**R**, 35**R**, 37**R**.
